# MS-TFNet: a multi-scale temporal feature fusion network for maize growth stage recognition

**DOI:** 10.3389/fpls.2026.1882260

**Published:** 2026-08-21

**Authors:** Su Wu, Zhihao Liu, Dongli Wu, Yijun Liu, Hanxiao Zhang, Mengxuan Liu, Dongzhe Tian, Guisheng Yin

**Affiliations:** 1 College of Computer Science and Technology, Harbin Engineering University, Harbin, China; 2 Henan Zhongyuan Photoelectric Measurement and Control Technology Co., Ltd., Zhengzhou, China; 3 Meteorological Observation Centre, China Meteorological Administration, Beijing, China

**Keywords:** attention mechanisms, field-based RGB imagery, maize growth stage recognition, multi-scale feature fusion, temporal feature embedding

## Abstract

Accurate maize growth stage recognition is critical for precision agricultural management, but it remains challenging due to subtle morphological differences between adjacent stages, small seedling targets, and substantial cross-year environmental variability. To address these challenges, this study proposes a Multi-Scale Temporal Feature Fusion Network, termed MS- TFNet, for image-level maize growth stage recognition using red-green-blue (RGB) images and acquisition-date information. The proposed network integrates three complementary components. First, a Multi-Scale Feature Extraction Module (MS) is introduced to capture maize structural variations at different receptive-field scales. Second, a Vegetation-Aware Convolutional Block Attention Module (VCBAM) is designed to adaptively refine RGB feature responses and improve the representation of maize-related visual cues under complex field backgrounds. Third, a Temporal Feature Module (TFM) encodes image acquisition dates using date-based sine–cosine temporal encoding, enabling phenological prior information to be incorporated into single-image growth stage classification. Experiments were conducted on a long-term field observation dataset containing 24,239 maize images collected over eight growing seasons and covering nine maize growth stages. The results show that MS-TFNet achieved a mean Overall Accuracy (OA) of 83.64%, outperforming the ResNet-50 baseline by 4.46 percentage points. Under a ±3-day temporal tolerance criterion, the recognition accuracy further increased to 93.76%, indicating improved robustness for practical crop monitoring scenarios. Overall, MS-TFNet provides an effective framework for maize growth stage recognition under complex field conditions by jointly exploiting multi-scale visual representation, vegetation-aware attention, and acquisition-date-based phenological priors.

## 1 Introduction

Accurate identification of crop growth stages is a fundamental prerequisite for precision agricultural management and plays a critical role in guiding key agronomic practices such as irrigation scheduling, fertilization, and pest and disease control (Li et al., 2010; Long et al., 2017; Shu et al., 2020). As one of the world’s major staple crops, maize has attracted increasing attention in terms of automated growth monitoring, which has become an important research direction in smart agriculture (Sharma et al., 2022). Conventional determination of crop growth stages mainly relies on manual field observations, which are labor-intensive, time-consuming, and difficult to scale to large agricultural areas (Bouman et al., 1996; van Ittersum et al., 2003; Omari et al., 2020). Consequently, the development of automated and intelligent growth stage recognition techniques has become essential for improving agricultural management efficiency (Ni et al., 2024; Sun et al., 2020).

With the rapid advancement of precision agriculture, the demand for automated crop monitoring systems has grown substantially. Although remote sensing and unmanned aerial vehicle (UAV) technologies have been applied to crop monitoring, they still suffer from limitations such as insufficient spatial resolution, inflexible revisit cycles, or high acquisition costs (Xuejun et al., 2023). In contrast, ground-based image analysis offers advantages including low cost, high spatial resolution, and real-time capability, making it particularly suitable for fine-grained field management (Kamilaris and Prenafeta-Boldú, 2018; Simonyan and Zisserman, 2014; Tan and Le, 2019). Nevertheless, such approaches remain challenged by limited data availability, complex field environments, and large intra-class variability (Quan et al., 2019).

Recent progress in deep learning has provided powerful tools for agricultural image analysis. Convolutional neural networks (CNNs) have been widely adopted in agricultural applications, including disease detection (Liu et al., 2024; Jung et al., 2024; Mustafa et al., 2024; Li et al., 2025b), yield prediction (Fei et al., 2023; Zhou et al., 2025), and crop growth stage classification (Li et al., 2025a; Schieck et al., 2023; Kim et al., 2025). A comprehensive review by Attri et al. (2023) highlights the strong capability of CNNs in feature extraction and pattern recognition. Classical architectures such as ResNet (He et al., 2016), VGG Simonyan and Zisserman (2014), and EfficientNet (Tan and Le, 2019) have demonstrated promising performance in crop image analysis; however, their high computational complexity and large data requirements remain significant challenges (Singh et al., 2021; Albahli, 2025). Existing studies primarily focus on extracting visual features from single images and often lack targeted designs to address the specific challenges of maize growth stage recognition, including confusion between adjacent stages, early-stage small-object detection, and pronounced spatio-temporal variability. As a result, multi-scale feature extraction and attention mechanisms have emerged as important research directions in this field (Won, 2020; Zheng et al., 2024; Tang et al., 2024).

Multi-scale feature extraction enables effective representation of image information across different receptive fields and is particularly important for distinguishing subtle morphological differences between growth stages. Lin et al. proposed the Feature Pyramid Network (FPN), which enhances multi-scale representations through a top-down pathway (Lin et al., 2017a), while He et al. introduced Spatial Pyramid Pooling (SPP) to improve adaptability to objects of varying scales (He et al., 2014). In terms of attention mechanisms, Hu et al. developed the Squeeze-and-Excitation Network (SENet) to enhance feature discrimination by adaptively reweighting channel responses (Hu et al., 2018), and Woo et al. proposed the Convolutional Block Attention Module (CBAM), which jointly models channel-wise and spatial attention to further improve feature representation (Woo et al., 2018). These mechanisms have demonstrated strong robustness in agricultural tasks such as crop phenotyping and field semantic segmentation (Koirala et al., 2019; Cai et al., 2023; Xiong et al., 2021), providing a theoretical basis for precise vegetation localization and feature enhancement.

Agricultural image datasets are often constrained by uneven growth stage durations, seasonal variations, climatic conditions, and acquisition equipment, leading to insufficient sample sizes and severe class imbalance. Data augmentation is therefore widely used to enhance model generalization. For example, generative adversarial networks (GANs) have been employed to expand crop image datasets and alleviate overfitting (Goodfellow et al., 2014), while sample-mixing strategies such as MixUp and CutMix have been introduced to improve model robustness (Hongyi et al., 2018; Yun et al., 2019). In addition, loss function design plays a crucial role in handling class imbalance. Focal Loss effectively mitigates the dominance of majority classes (Lin et al., 2017b), whereas contrastive learning losses enhance intra-class compactness and inter-class separability, and have been successfully applied in agricultural phenotype recognition (Chen et al., 2020).

Temporal information is relevant for crop growth stage identification, as crop development follows seasonal patterns, and similar visual appearances may correspond to different growth stages at different times. In recent years, multimodal learning has attracted increasing attention in agricultural monitoring. For instance, time-series remote sensing imagery has been used to improve crop classification accuracy (Rußwurm and Körner, 2018), and the fusion of meteorological data with visual features has been explored for yield prediction. However, most existing studies focus on long-term temporal modeling, while effective incorporation of acquisition date information into single-image-based growth stage recognition remains relatively underexplored.

Despite the notable progress achieved by deep learning-based approaches, maize growth stage recognition from field images still faces several critical challenges:

- High morphological similarity between adjacent stages, such as between seeding and seedling emergence, or between jointing and tasseling, where visual differences are subtle and prone to misclassification (Yang et al., 2025);
- Difficulty in early-stage small-object detection, as seedlings occupy a small proportion of the image and are easily affected by background clutter, illumination variation, and occlusion, leading to insufficient feature representation (Dalal and Mittal, 2025; Feng et al., 2025a);
- Strong spatio-temporal variability, where differences in year, field location, and management practices result in substantial variations in plant color, texture, and growth patterns, thereby degrading cross-domain generalization performance (Khan et al., 2025; Shi et al., 2025).

Existing studies primarily focus on visual feature extraction from single images, while insufficient attention has been paid to the integration of temporal priors and domain-specific feature enhancement mechanisms. As a result, current methods often struggle to maintain stable performance across different growth stages and environmental conditions.

To address these challenges, this study proposes a maize growth stage recognition framework that integrates multi-scale feature extraction, vegetation-enhanced attention, and temporal priors. Multi-scale features are used to capture structural variations across growth stages, the attention mechanism enhances vegetation-related responses, and temporal information provides phenological constraints to improve prediction consistency. Overall, the proposed MS-TFNet improves the accuracy and robustness of maize growth stage recognition, providing a generalizable solution for crop growth monitoring in complex field environments.

## 2 Methodology

### 2.1 Dataset

**Figure 1:**
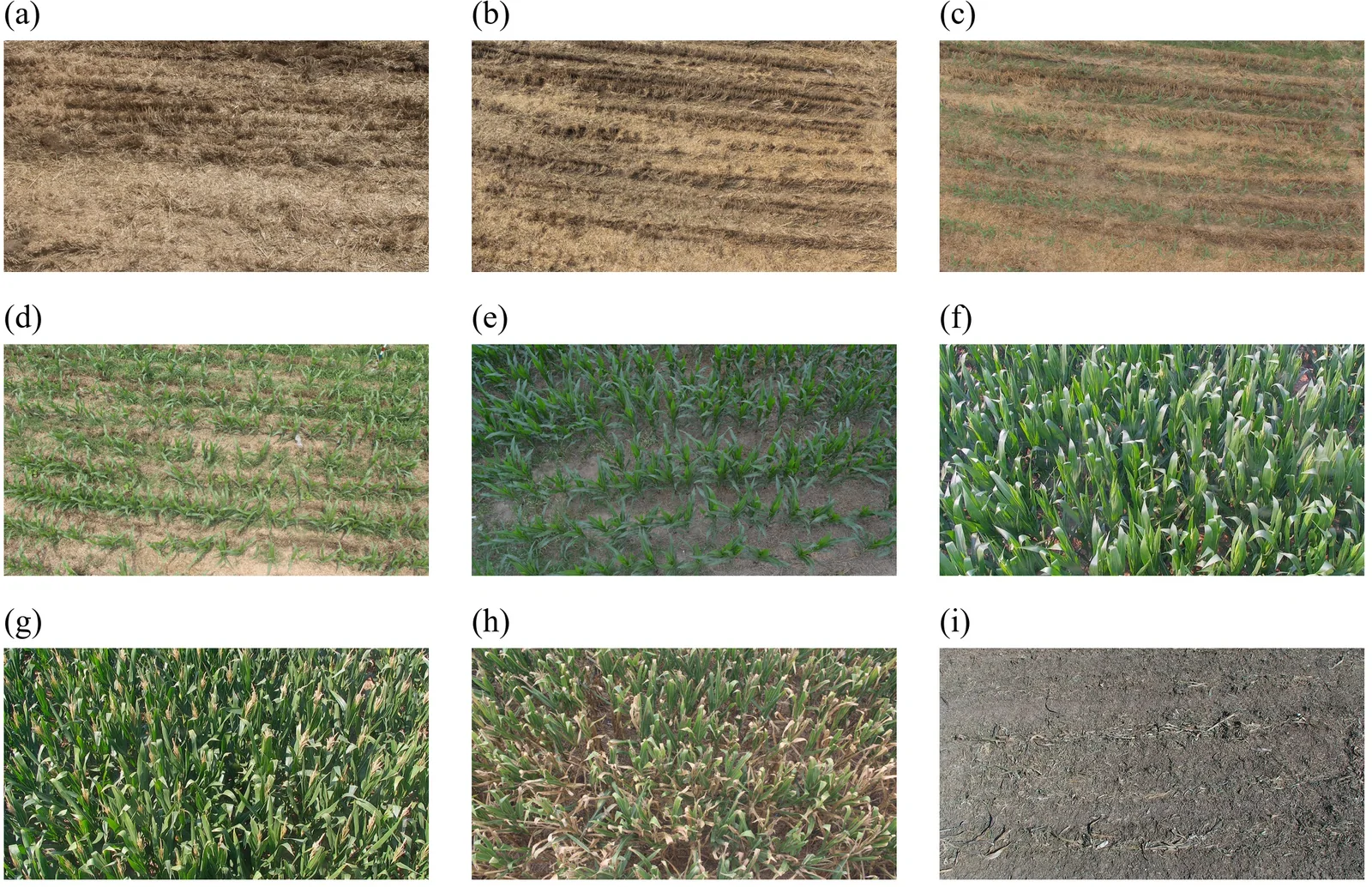
Representative field images of different maize growth stages used in this study. **(a)** Stage 11; **(b)** Stage 21; **(c)** Stage 31; **(d)** Stage 41; **(e)** Stage 61; **(f)** Stage 71; **(g)** Stage 81; **(h)** Stage 91; **(i)** Stage 99.

The maize growth stage monitoring dataset used in this study is a large-scale field image dataset with rich temporal and spatial coverage, designed to support automated recognition and classification of maize growth stages. The data were collected from six fixed observation stations (O3543, O3544, O3545, O6965, O8562, and O8563) belonging to the China Agricultural Meteorological Automatic Monitoring System. Image acquisition spanned from 2016 to 2023, covering eight complete maize growing seasons. Each observation site was equipped with a high-resolution field camera with a spatial resolution of 2560 × 1920 pixels. Images were automatically captured once per hour during daylight hours (09:00–18:00) each day, resulting in a total of 24,239 raw field images. This acquisition strategy ensured continuous monitoring of maize growth dynamics under natural field conditions. The dataset covers the complete maize growth cycle from seeding to post-harvest and includes nine key growth stages. These stages are defined as seeding (Stage 11, characterized by bare soil with no visible seedlings), seedling emergence (Stage 21), three-leaf (Stage 31), seven-leaf (Stage 41), jointing (Stage 61), tasseling (Stage 71), milk (Stage 81), maturity (Stage 91), and post-harvest (Stage 99, showing harvested fields with minimal vegetation). Representative examples are shown in **Figure 1**.

The dataset is organized in a hierarchical directory structure to facilitate efficient indexing and analysis. The directory format follows: *station ID/year/growth stage/timestamped image file*. **Table 1** summarizes the original sample statistics before data augmentation. Three station-year subsets were selected and held out as independent cross-year test sets, and the remaining data were used for model development. Data augmentation was applied only to the training subset of the model-development data, rather than to the independent test sets.

**Table 1:** Dataset composition and sample statistics for each growth stage.

Stage code	11	21	31	41	61	71	81	91	99
Stage Name	Seeding	Seedling Emergence	Three-leaf	Seven-leaf	Jointing	Tasseling	Milk	Maturity	Post-harvest
Samples	1221	344	1953	2256	4037	5034	5178	2260	1956
Ratio (%)	5.04	1.42	8.06	9.31	16.65	20.77	21.36	9.32	8.07
Avg. Duration (days)	8	4	12	14	25	28	30	16	18

### 2.2 Problem formulation

The objective of this study is to classify the maize growth stage of a field image using visual information and acquisition-date metadata. Each sample is defined as (*I_i_,d_i_,y_i_*), where $I_{i}\in {\mathbb{R}}^{H\times W\times 3}$ denotes a single RGB field image, *d_i_* denotes the corresponding acquisition date, and *y_i_* denotes the ground-truth growth-stage label. The label *y_i_* belongs to one of nine maize growth stages, including Stage 11, Stage 21, Stage 31, Stage 41, Stage 61, Stage 71, Stage 81, Stage 91, and Stage 99.

The proposed model learns a mapping from the input image and its acquisition date to a probability distribution over the nine growth-stage classes as defined in Equation (1):

(1) $${\hat{p}}_{i}=f_{\theta }(I_{i},d_{i})$$

where ${\hat{p}}_{i}\in {\mathbb{R}}^{9}$ represents the predicted class-probability vector, and *θ* denotes the learnable model parameters. The final predicted growth stage is determined by the class with the highest probability as defined in Equation (2):

(2) $${\hat{y}}_{i}=\arg\;\underset{k}{\max}{\hat{p}}_{i,k}$$

It should be noted that the task is formulated as single-image classification with temporal metadata, rather than sequence-based classification. The acquisition date is used as auxiliary phenological information through the TFM, while the image itself remains the primary input for visual feature extraction.

### 2.3 Data preprocessing and augmentation

Before model training, all RGB field images were resized to 512 × 512 pixels and normalized to ensure a consistent input format. Data augmentation was applied only to the training set and was not applied to the internal validation set and the independent station-year test subsets.

The dataset exhibits pronounced imbalance in both spatial distribution and class composition. To reduce the influence of class imbalance and improve the robustness of feature learning, a hierarchical data augmentation strategy was adopted. The augmentation operations included:

- Basic augmentation, such as brightness and contrast adjustment, hue-saturation-value (HSV) transformation, geometric transformation, and random occlusion;
- Distribution-aware augmentation, where minority classes, such as seedling emergence and post-harvest stages, were augmented with higher intensity and frequency.

**Figure 2:**
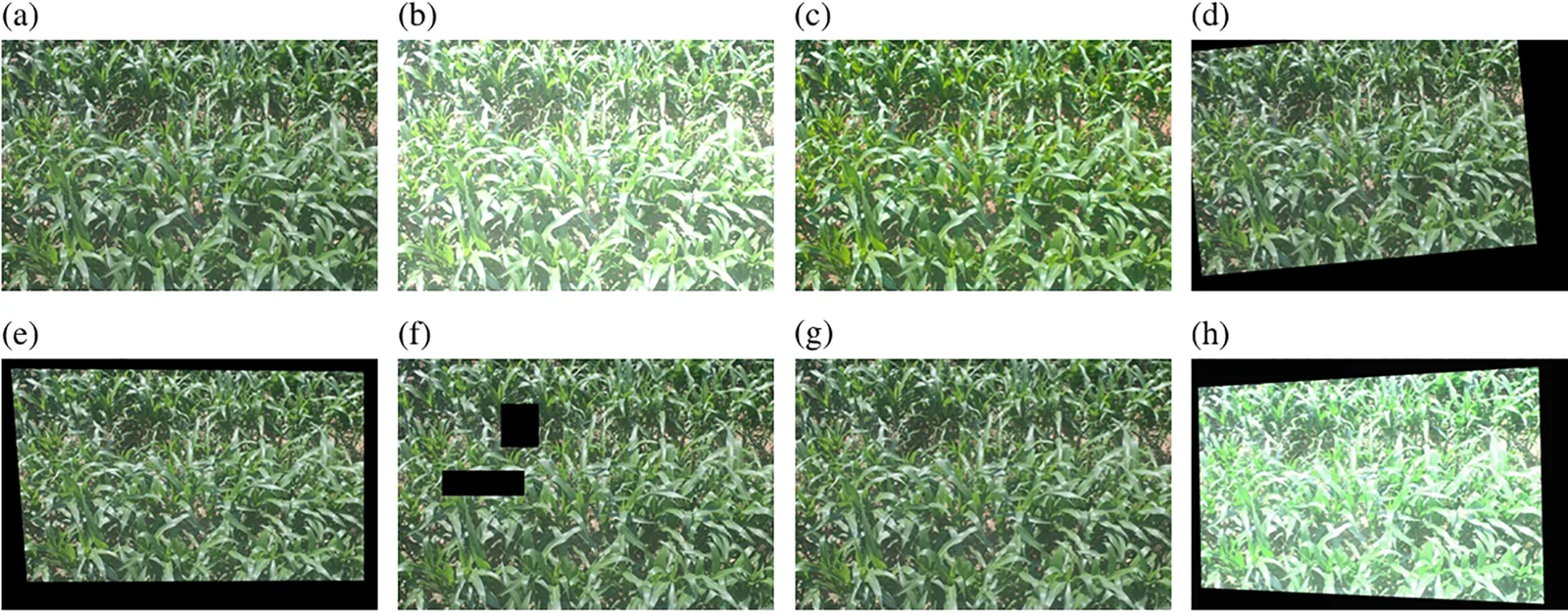
Visualization of data augmentation strategies used in this study: **(a)** original image; **(b)** brightness and contrast adjustment; **(c)** color space transformation; **(d)** geometric transformation; **(e)** perspective transformation; **(f)** dropout enhancement; **(g)** noise and blur augmentation; **(h)** comprehensive augmentation.

Following this strategy, class imbalance was mitigated. The dataset size was expanded to approximately 32,000 training images, with each growth stage containing more than 2,000 samples. Representative augmentation examples are provided in **Figure 2**. Data augmentation was applied only to the training set after excluding the held-out test subsets.

### 2.4 Overview of MS-TFNet

**Figure 3:**
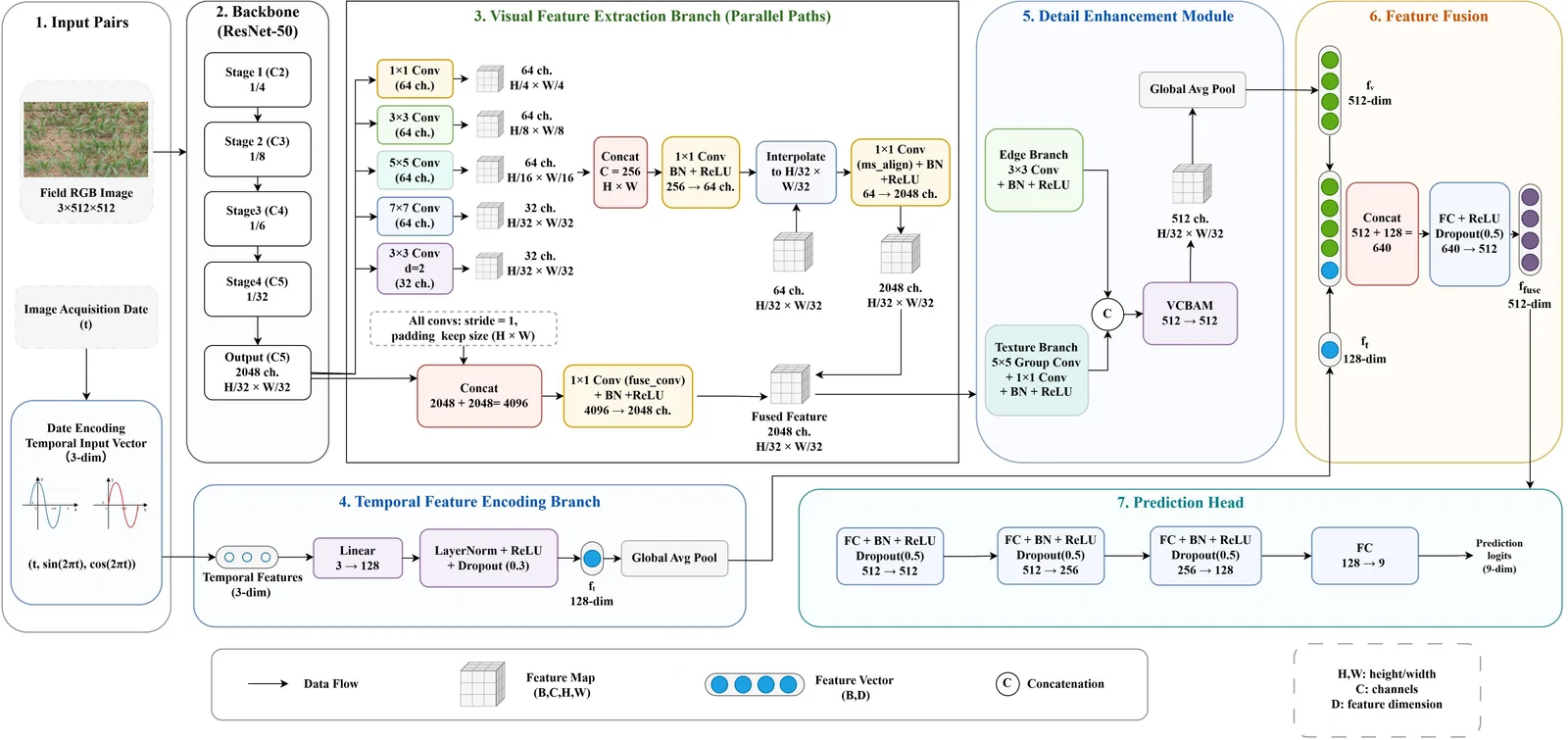
Overall architecture of the proposed MS-TFNet. The model receives an RGB field image and its acquisition date as inputs. The visual branch extracts a 512-dimensional global visual feature using ResNet-50, MS module, and VCBAM-based detail enhancement. In parallel, the temporal branch converts the acquisition date into a three-dimensional temporal input vector, [*t*,sin(2*πt*),cos(2*πt*)], and projects it into a 128-dimensional temporal embedding. The visual feature and temporal embedding are concatenated to form a 640-dimensional fused feature vector, which is subsequently passed.

A maize growth stage recognition model integrating multi-scale feature extraction, VCBAM, and temporal feature fusion is proposed in this study. The overall architecture of the model is illustrated in **Figure 3**. ResNet-50 is adopted as the backbone network to extract high-level semantic features from RGB field images.

In parallel, a multi-scale feature extraction branch is introduced and directly applied to the original input images to capture crop texture and structural information at different spatial scales. The extracted multi-scale features are aligned with the backbone features in both spatial resolution and channel dimension, and then fused to enhance complementary representations. Subsequently, a detail enhancement module and the proposed VCBAM are employed to suppress background interference and emphasize crop-related discriminative features.

On top of the visual feature representation, a late-fusion strategy is adopted to incorporate temporal information. Specifically, the encoded temporal features are concatenated with the global image features before classification. The fused representation is finally fed into the classification head to predict nine maize growth stages.

### 2.5 Multi-scale feature extraction module

Maize plants exhibit substantial morphological changes throughout the vegetative and reproductive growth stages. During the early stages, seedlings occupy only a small proportion of the field image and are easily affected by soil background, shadows, weeds, and other field factors. As maize develops, leaves, stems, tassels, and canopy structures gradually increase in size and complexity. In addition, adjacent growth stages often show similar visual appearances and overlapping phenotypic characteristics. These factors make it difficult for features extracted at a single spatial scale to adequately represent maize growth stages across the entire growth cycle.

Multi-scale feature extraction provides an effective way to integrate complementary spatial information from different receptive fields. Parallel convolutional branches with different kernel sizes can capture visual patterns at multiple scales while preserving local details (Szegedy et al., 2015). Multi-kernel structures have also been used in agricultural image analysis to improve the representation of targets with large variations in size and field appearance. Therefore, a MS Module is introduced as a parallel branch of the backbone network to enhance the spatial representation of maize plants at different growth stages.

The proposed module contains four standard convolutional branches with kernel sizes of 1 × 1, 3 × 3, 5 × 5, and 7 × 7, together with a 3 × 3 dilated convolutional branch with a dilation rate of *r* = 2 (Yu and Koltun, 2016). All branches process the same input feature map using different receptive-field sizes. The 1 × 1 convolution integrates channel-wise information in the input feature map while preserving the spatial resolution. The 3 × 3, 5 × 5, and 7 × 7 convolutions progressively enlarge the receptive field, allowing the module to represent spatial patterns at different scales.

The dilated convolutional branch further expands the effective receptive field without using a larger conventional kernel. By inserting spaces between kernel elements, dilated convolution increases contextual coverage while maintaining the spatial resolution of feature maps. The combination of standard and dilated convolutions is intended to provide both local visual details and broader contextual information for maize growth-stage recognition.

The feature maps generated by the five branches are concatenated along the channel dimension. A subsequent 1 × 1 convolution, followed by Batch Normalization (BN) and a Rectified Linear Unit (ReLU) activation function, is used to fuse the concatenated features into a unified 64-channel representation. The resulting multi-scale features are then aligned with the backbone features in spatial resolution and channel dimension before being integrated into the subsequent feature-refinement process.

By combining features extracted from multiple receptive-field scales, the proposed module provides complementary spatial information for recognizing maize plants with different sizes and structural characteristics. The contribution of this module to growth-stage classification is quantitatively evaluated through the ablation experiments presented in Section 3.3.

### 2.6 Vegetation-aware convolutional block attention module

**Figure 4:**
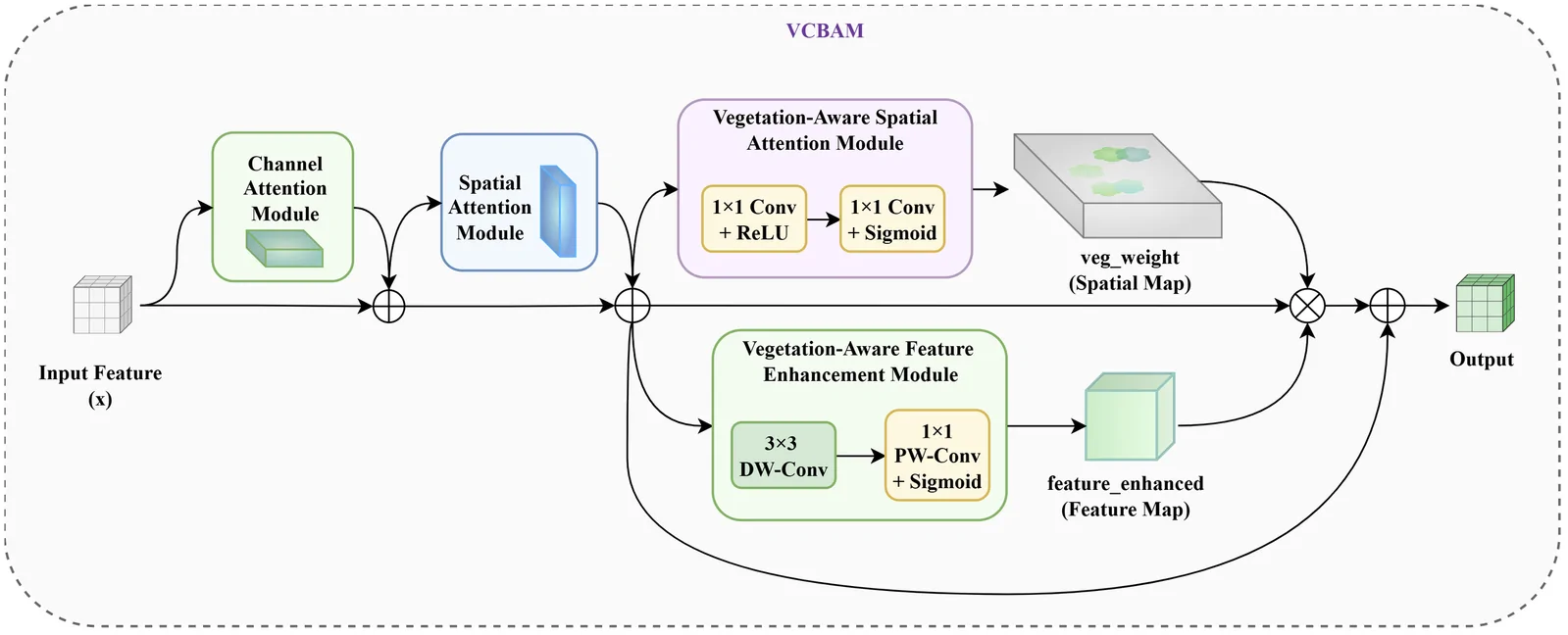
Structure of the proposed Vegetation-Aware Convolutional Block Attention Module (VCBAM). The module first applies the standard channel and spatial attention operations of CBAM, and then introduces two vegetation-aware refinement branches. The upper branch generates a vegetation-aware spatial response map *V_s_*, while the lower branch generates a feature enhancement weight *G_w_*. The two learned responses are combined with the CBAM-refined feature map to enhance crop-related feature responses and reduce background responses.

Field-based maize growth stage recognition is challenged by complex backgrounds, large variations in target scale, and weak discriminative visual cues, especially during early growth stages when seedlings are easily confused with bare soil. Attention mechanisms have been widely used to recalibrate feature responses and enhance task-relevant representations in convolutional neural networks (Woo et al., 2018). To address these issues, we propose VCBAM, a domain-oriented attention mechanism designed for RGB field images. The structure of the proposed VCBAM is shown in **Figure 4**.

Building upon the classical CBAM, the proposed module introduces an additional vegetation-aware refinement stage after the standard channel and spatial attention modules. The refinement stage is designed to learn crop-related spatial responses and feature enhancement weights directly from RGB feature maps. By adaptively refining vegetation-related feature responses, the module aims to improve the discriminative representation of maize plants under complex field conditions. This design is particularly useful for early growth stages, where maize seedlings occupy a small proportion of the image and are easily confused with soil background, shadows, and other field interference.

To adapt the attention mechanism to RGB field images, a data-driven strategy is employed to learn vegetation-sensitive responses directly from intermediate RGB feature representations. Based on this design, a vegetation-aware spatial response map is constructed as defined in Equation (3):

(3) $$V_{s}=\sigma (W_{v}^{\left(2\right)}(\text{ReLU}(W_{v}^{\left(1\right)}(F_{cs}))))$$

where *F_cs_* denotes the CBAM-refined feature map; $W_{v}^{\left(1\right)}$ and $W_{v}^{\left(2\right)}$ represent the learnable parameters of two successive convolution layers; ReLU(·) denotes the rectified linear activation function; and *σ*(·) denotes the Sigmoid activation function. The generated tensor $V_{s}\in {\mathbb{R}}^{B\times 1\times H\times W}$ represents the vegetation-aware spatial response map.

In parallel, a feature enhancement weight is generated to capture joint channel–spatial correlations as defined in Equation (4):

(4) $$G_{w}=\sigma ({\text{Conv}}_{1\times 1}({\text{DWConv}}_{3\times 3}(F_{cs})))$$

where DWConv_3×3_(·) denotes depthwise convolution for local texture extraction. The generated tensor $G_{w}\in {\mathbb{R}}^{B\times C\times H\times W}$ represents the learned channel–spatial feature enhancement weights.

The final refined feature is obtained by integrating the vegetation-aware spatial response and feature enhancement weight with the CBAM-refined feature as defined in Equation (5):

(5) $$F_{out}=F_{cs}⊙(1+V_{s}\cdot G_{w})$$

where ⊙ denotes element-wise multiplication, and *V_s_* is broadcast along the channel dimension to match the size of *G_w_*. The multiplicative enhancement term enables adaptive amplification of vegetation-sensitive regions while preserving stable feature propagation.

By integrating vegetation-aware cues with attention-refined features, the proposed module aims to improve the representation of crop-related visual cues under complex field backgrounds. This mechanism is particularly useful for recognizing small maize targets during early growth stages, where plant structures are visually weak and easily affected by soil, shadows, and other field interference. The effectiveness of VCBAM is evaluated through comparisons with other attention mechanisms and ablation experiments in Sections 3.2 and 3.3.

### 2.7 Temporal feature module

Maize growth stage recognition is not determined solely by visual appearance, but is also closely associated with seasonal phenological progression. In field images, adjacent growth stages may show similar visual characteristics, especially around stage-transition periods. Therefore, the acquisition date of an image can provide useful auxiliary information for distinguishing visually similar growth stages. Temporal and phenological information has been widely used in crop monitoring to describe seasonal development patterns and improve the representation of crop growth dynamics (Jönsson and Eklundh, 2004).

In this study, TFM converts the acquisition date of each image into a three-dimensional temporal input vector and subsequently projects it into a learnable 128-dimensional temporal embedding. The proposed TFM does not model a sequence of consecutive images. Instead, each sample consists of a single RGB image and its corresponding acquisition-date metadata. The resulting temporal embedding is used as auxiliary phenological information and is fused with the global visual feature before classification.

For each image, the acquisition date is represented by its calendar month and day. To obtain a continuous temporal descriptor, the date is first converted into a normalized intra-year position as defined in Equation (6):

(6) $$t=\frac{(\text{month}-1)\times 31+\text{day}}{12\times 31}$$

where month and day denote the acquisition month and day of the image, respectively. The denominator 12 × 31 is used to normalize the encoded date into a fixed annual scale. This formulation provides a simple and consistent temporal representation for all images, allowing the model to use relative seasonal position as auxiliary information.

To further represent the periodic nature of crop growth within a year, sine-cosine encoding is applied to the normalized temporal position as defined in Equation (7):

(7) s=sin (2πt), c=cos (2πt)

To represent both the relative seasonal position and annual periodicity, the normalized intra-year position is combined with its sine and cosine components to form a three-dimensional temporal input vector as defined in Equation (8):

(8) $$T_{input}=[t,s,c]$$

where *s* and *c* denote the sine and cosine components of the normalized date, respectively. This representation preserves both the relative position in the growing season and the cyclic property of annual crop development.

The three-dimensional temporal input vector is subsequently projected into a learnable 128-dimensional temporal embedding through a lightweight encoder consisting of a linear layer, layer normalization, ReLU activation, and dropout as defined in Equation (9):

(9) $$T_{feat}=\text{Dropout}(\text{ReLU}(\text{LayerNorm}(W_{t}T_{input}+b_{t})))$$

where $W_{t}\in {\mathbb{R}}^{128\times 3}$, $b_{t}\in {\mathbb{R}}^{128}$, and $T_{\text{feat}}\in {\mathbb{R}}^{128}$ denotes the learned temporal embedding.

After the visual branch extracts the global image feature, the temporal embedding is concatenated with the visual feature as defined in Equation (10):

(10) $$F_{fused}=\text{Concat}(F_{img},T_{feat})$$

The fused representation is further transformed by a fully connected projection layer before being fed into the final classifier as defined in Equation (11):

(11) $$F_{out}=\text{Dropout}(\text{ReLU}(\text{LayerNorm}(W_{f}F_{fused}+b_{f})))$$

where $F_{\text{img}}\in {\mathbb{R}}^{512}$, $T_{\text{feat}}\in {\mathbb{R}}^{128}$, and $F_{\text{fused}}\in {\mathbb{R}}^{640}$.

By incorporating acquisition-date information into the classification process, TFM provides a lightweight phenological prior for maize growth stage recognition. The effectiveness of this module is evaluated through ablation experiments and temporal tolerance analysis in Sections 3.3.

### 2.8 Experimental settings

To ensure a fair comparison among different models, all experiments were conducted under a unified experimental setting. This section describes the data partition strategy, evaluation metrics, and implementation details used in this study.

#### 2.8.1 Experimental design and data partition

To ensure a clear distinction between model development and independent evaluation, the dataset was divided into a model-development set and three independent station-year test subsets. The model- development set was used for model training and internal validation, where the internal validation split was used only for monitoring training convergence and selecting the best checkpoint. The three station-year subsets from 2020, 2021, and 2022 were held out from model development and used only for independent cross-year testing.

Each input sample consisted of a single RGB field image and its corresponding acquisition date. All RGB images were resized to 512 × 512 pixels before being fed into the network. The acquisition date was encoded as a three-dimensional temporal feature vector, [*t*,sin(2*πt*),cos(2*πt*)], as described in Section 2.7. This input setting was consistently used for all compared models.

The composition of the three independent station-year test subsets is shown in **Table 2**. Specifically, the independent test subsets contained 1035 images from 2020, 832 images from 2021, and 588 images from 2022. The three subsets were collected under different station-year conditions and were used to evaluate whether the trained models could generalize to unseen cross-year field conditions.

**Table 2:** Composition of the three independent station-year test subsets used for cross-year evaluation.

Station-year test subset	Total	Stage 11	Stage 21	Stage 31	Stage 41	Stage 61	Stage 71	Stage 81	Stage 91	Stage 99
O6965-2020	1035	40	16	111	128	150	166	226	47	151
O6965-2021	832	84	0	76	107	80	201	202	9	73
O8563-2022	588	44	11	64	113	92	96	100	61	7

O6965 and O8563 denote field monitoring station identifiers. The three station-year subsets were used only as independent cross-year test sets and were not involved in model training or internal validation. Stage 21 was absent from the O6965–2021 independent test subset.

#### 2.8.2 Evaluation metrics

OA was used as the primary evaluation metric. OA measures the proportion of correctly classified samples among all samples and is defined in Equation (12):

(12) $$OA=\frac{N_{correct}}{N}$$

where *N_correct_* denotes the number of correctly classified samples and *N* denotes the total number of samples.

To further evaluate classification performance under imbalanced class distributions, Precision, Recall, and F1-score were also calculated. For the k-th growth-stage class, Precision, Recall, and F1-score are defined in Equations (13–15), respectively:

(13) $$Precision_{k}=\frac{TP_{k}}{TP_{k}+FP_{k}}$$

(14) $$Recall_{k}=\frac{TP_{k}}{TP_{k}+FN_{k}}$$

(15) $$F1_{k}=\frac{2\times Precision_{k}\times Recall_{k}}{Precision_{k}+Recall_{k}}$$

where *TP_k_*, *FP_k_*, and *FN_k_* denote the numbers of true positives, false positives, and false negatives for class *k*, respectively. Macro-averaged metrics were obtained by averaging the corresponding class-wise metrics over all growth-stage classes as defined in Equation (16):

(16) $$Macro-F1=\frac{1}{K}\sum_{k=1}^{K}F1_{k}$$

where *K* denotes the number of maize growth-stage classes. In this study, *K* = 9.

#### 2.8.3 Implementation details

All models were implemented using the PyTorch framework. The AdamW optimizer was used for model optimization, with an initial learning rate of 1 × 10^−4^ and a batch size of 32. A cosine annealing learning-rate scheduler with warm restarts was adopted to improve convergence stability.

To alleviate the influence of class imbalance, weighted Focal Loss was used as the loss function, where class weights were set inversely proportional to the number of samples in each growth-stage class. During training, model performance was monitored on the internal validation set, and the checkpoint with the highest validation accuracy was selected for final evaluation on the independent cross-year test sets.

All images were resized to 3×512×512 as model input. Computational complexity was evaluated using the number of learnable parameters, floating-point operations (FLOPs), inference speed, and model size. FLOPs were calculated for a single forward pass with an input size of 3 × 512 × 512. Inference speed was measured as frames per second (FPS) under the same hardware and batch-size setting. Model size refers to the storage size of the saved model weight file on disk.

## 3 Results

### 3.1 Backbone network comparison

**Figure 5:**
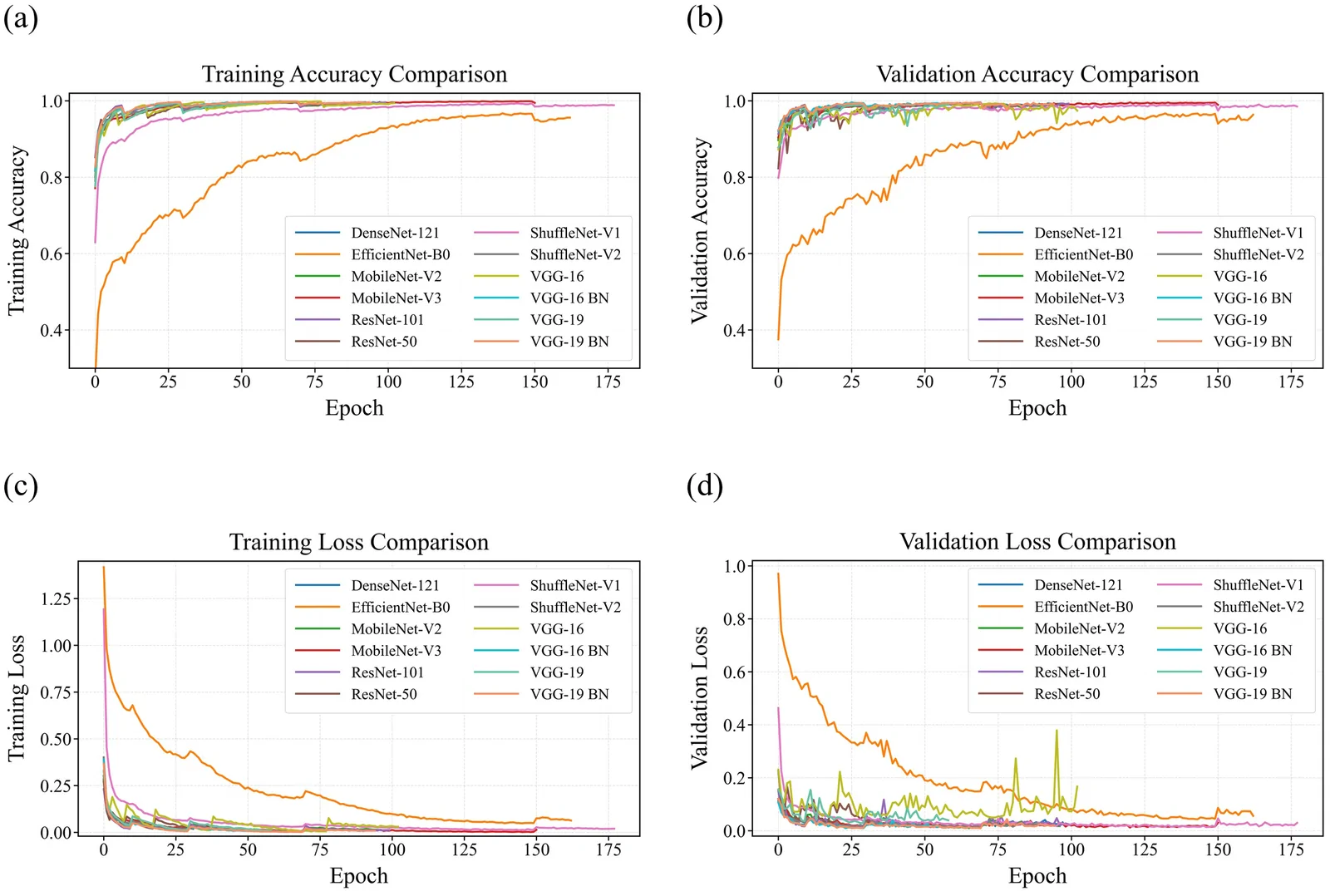
Training and internal-validation curves of different backbone networks: **(a)** training accuracy; **(b)** validation accuracy; **(c)** training loss; **(d)** validation loss. The validation curves refer to the validation split used during model training, whereas the independent cross-year test results are reported in **Table 3**.

To select an appropriate backbone network for maize growth stage recognition, twelve representative CNN architectures were evaluated under the same input setting, training strategy, and data augmentation configuration, including the VGG series, ResNet series, MobileNet series, EfficientNet-B0, ShuffleNet series, and DenseNet-121. The training and internal-validation curves of these backbone networks are shown in **Figure 5**. These curves were used to examine the training behavior of different models, whereas the final backbone selection was based on their performance on independent cross-year test sets.

Under the unified training configuration, most backbone networks gradually converged in terms of training and internal-validation accuracy and loss, indicating that the optimization strategy was generally effective. However, satisfactory convergence on the model-development data did not necessarily translate into robust generalization under unseen station-year conditions. Therefore, the final backbone selection was based primarily on performance on the three independent station-year test subsets. **Table 3** reports the OA for each test subset, together with mean OA, mean Macro-F1, and computational complexity.

**Table 3:** Performance and computational complexity comparison of different backbone networks on three independent cross-year test sets.

Model	2022 Test OA (%)	2021 Test OA (%)	2020 Test OA (%)	Mean OA (%)	Mean Macro-F1 (%)	Params (M)	FLOPs (G)	FPS	Size (MB)
VGG-16	72.62	77.40	81.64	77.22	70.12	14.72	80.18	21.50	58.88
VGG-16 BN	74.66	75.96	76.43	75.68	64.68	14.73	80.46	19.89	58.91
VGG-19	72.62	**83.29**	81.16	79.02	71.79	20.03	101.92	17.28	80.12
VGG-19 BN	**75.51**	78.97	77.68	77.39	72.18	20.04	102.23	16.15	80.16
ResNet-50	73.47	79.33	**84.73**	**79.18**	73.45	23.53	21.59	46.79	94.11
ResNet-101	73.64	83.05	80.39	79.03	**75.02**	42.52	41.09	26.88	170.07
MobileNetV2	75.17	81.49	79.03	78.56	69.13	2.24	1.70	148.58	8.94
MobileNetV3	65.14	73.20	81.93	73.42	65.64	2.98	1.21	181.77	11.92
EfficientNet-B0	57.82	63.94	69.47	63.74	61.79	4.02	2.01	103.06	16.08
ShuffleNetV1	73.30	75.84	81.74	76.96	72.47	**1.26**	**0.79**	**227.57**	**5.05**
ShuffleNetV2	74.66	79.93	80.29	78.29	68.40	5.36	3.11	163.50	21.45
DenseNet-121	72.79	83.17	80.58	78.85	67.13	6.96	15.13	35.85	27.85

Mean OA and mean Macro-F1 were calculated by averaging the corresponding metrics across the 2020, 2021, and 2022 independent test sets. Params (M) denotes the number of learnable parameters in millions. FLOPs (G) denotes floating-point operations in billions for a single forward pass with an input image size of 3×512×512. FPS denotes the number of images processed per second during inference under the same hardware and batch-size setting. Size (MB) denotes the storage size of the saved model weight file on disk. Bold values indicate the best result in each column.

As shown in **Table 3**, ResNet-50 achieved the highest mean OA among all compared backbone networks, with a value of 79.18%. Although VGG-19 obtained a higher OA than ResNet-50 on the 2021 test set, its mean OA was slightly lower than that of ResNet-50 and its computational cost was substantially higher. ResNet-101 achieved the highest mean Macro-F1. However, it required more parameters, a larger model size, and lower inference speed than ResNet-50. Lightweight networks, such as MobileNetV2, MobileNetV3, and ShuffleNet variants, achieved higher inference speed or smaller model size, but their mean OA or mean Macro-F1 values were lower than those of ResNet-50. Therefore, considering the requirements of subsequent model construction and edge-oriented deployment, ResNet-50 was selected as the backbone network because it provided a favorable trade-off among mean OA, mean Macro-F1, computational complexity, model size, and inference efficiency. This selection was not based on the best performance in a single year or a single metric. Additional macro-averaged metrics, including mean Macro-Precision, mean Macro-Recall, and mean Macro-F1, are provided in **Supplementary Table 1**.

### 3.2 Effectiveness analysis of attention mechanisms

To evaluate the influence of different attention mechanisms on maize growth stage recognition, SE, ECA, CBAM, and the proposed VCBAM were integrated into the ResNet-50 backbone under the same training and testing settings. The classification results on the three independent cross-year test sets are summarized in **Table 4**.

**Table 4:** Attention mechanism performance across three independent years.

Attention module	2022 Test OA (%)	2021 Test OA (%)	2020 Test OA (%)	Mean OA (%)
Baseline (ResNet-50)	73.47	79.33	84.73	79.18
SE	75.00	**81.25**	82.51	79.59
ECA	**84.52**	67.67	84.25	78.81
CBAM	79.59	71.39	83.29	78.09
VCBAM (Ours)	75.85	80.29	**87.83**	**81.32**

The results were calculated on three independent cross-year test sets from 2022, 2021, and 2020. SE, ECA, CBAM, and VCBAM denote Squeeze-and-Excitation, Efficient Channel Attention, Convolutional Block Attention Module, and the proposed Vegetation-Aware Convolutional Block Attention Module, respectively. The bold values indicate the best performance among all compared methods.

As shown in **Table 4**, the introduction of attention mechanisms changed the cross-year classification performance to different degrees. SE improved the mean OA from 79.18% to 79.59%, while ECA and CBAM showed lower mean OA than the baseline under the current cross-year setting. The proposed VCBAM achieved the highest mean OA of 81.32%, which was 2.14 percentage points higher than the ResNet-50 baseline. However, the performance advantage of each attention mechanism was not uniform across all individual test years. For example, SE achieved the highest OA on the 2021 test set, ECA achieved the highest OA on the 2022 test set, and VCBAM achieved the highest OA on the 2020 test set and the best mean performance across the three years.

Different attention mechanisms exhibited distinct cross-year performance patterns, which may be related to their feature-refinement strategies. SE and ECA mainly recalibrate channel-wise feature responses and can enhance informative semantic channels, but they provide limited spatial localization under complex field backgrounds. CBAM further incorporates spatial attention, enabling the model to capture spatially informative regions; however, its activation may still extend to surrounding soil, shadows, or senescent residues when these regions are visually correlated with maize growth stages. VCBAM introduces an additional vegetation-aware refinement stage, which encourages the model to emphasize crop-related regions while reducing background responses. The year-wise variation in **Table 4** indicates that the effectiveness of an attention mechanism depends not only on its architectural design, but also on the visual distribution of the corresponding test year, including illumination, canopy coverage, stage composition, and background conditions.

**Figure 6:**
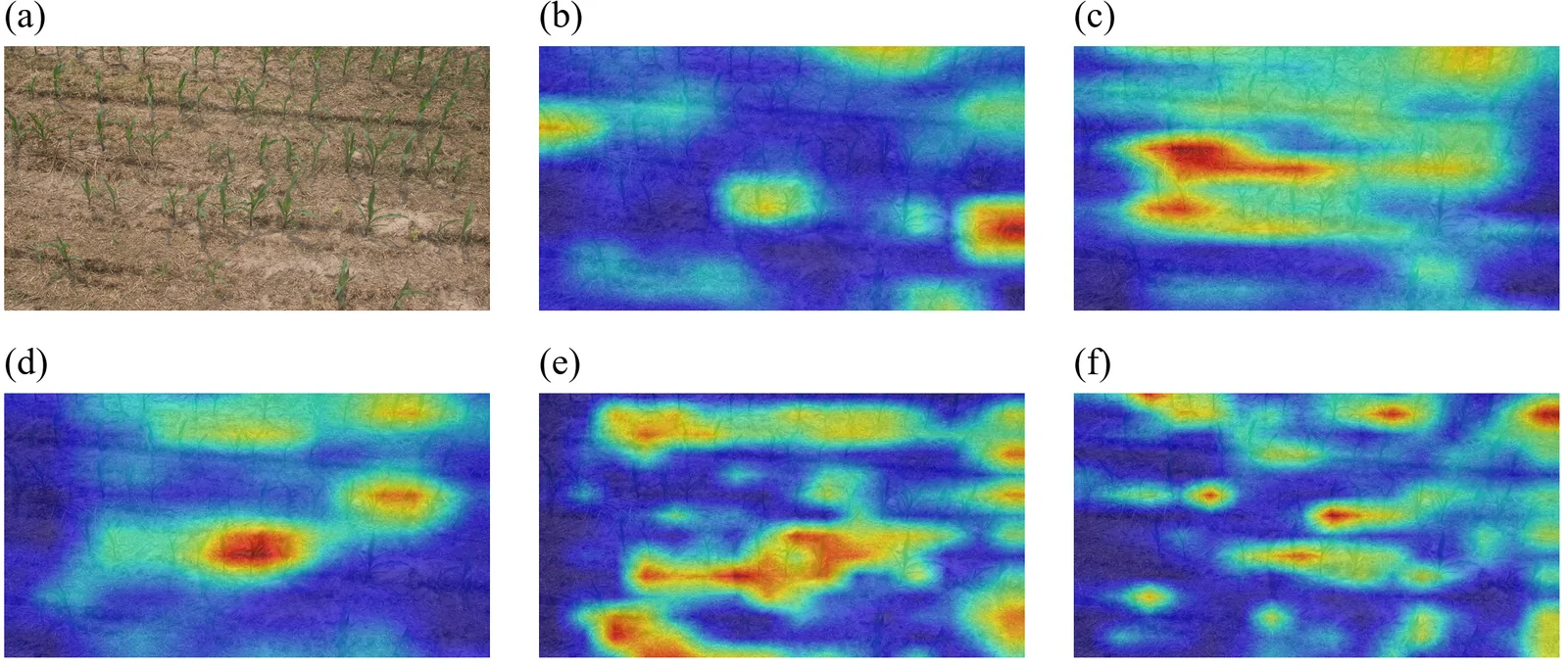
Grad-CAM visualization results for different attention mechanisms at Stage 41. **(a)** original image; **(b)** ResNet-50 baseline; **(c)** SE; **(d)** Efficient Channel Attention (ECA); **(e)** CBAM; **(f)** VCBAM. Warmer colors indicate regions with stronger model responses. Additional Grad-CAM visualization results for other maize growth stages are provided in **Supplementary Figure 1**.

Gradient-weighted Class Activation Mapping (Grad-CAM) was further used to compare the feature responses of different attention mechanisms, as shown in **Figure 6**. The baseline ResNet-50 produced relatively dispersed activation regions. SE and ECA enhanced feature responses through channel recalibration, but their spatial localization remained less concentrated under complex field backgrounds. CBAM introduced spatial attention and generated broader activation regions around maize plants and surrounding field areas. In comparison, VCBAM produced relatively more compact responses around crop-related regions, indicating that the vegetation-aware refinement stage helped enhance maize-related visual cues. The stage-wise Grad-CAM comparisons in **Supplementary Figure S1** further illustrate that the activation patterns of different attention mechanisms vary across maize growth stages.

To further examine the influence of exposed soil background on model recognition during early growth stages, an additional soil-background masking experiment was conducted. A vegetation mask was generated from the RGB image using green-dominance information, and non-vegetation regions, including soil and straw residues, were suppressed before Grad-CAM visualization. As shown in **Supplementary Figure S2**, the Grad-CAM responses changed after soil-background masking, indicating that exposed soil and straw residues can provide stage-related contextual cues for early growth-stage recognition. In other words, the exposed soil background may play an auxiliary role in recognizing early maize growth stages.

**Figure 7:**
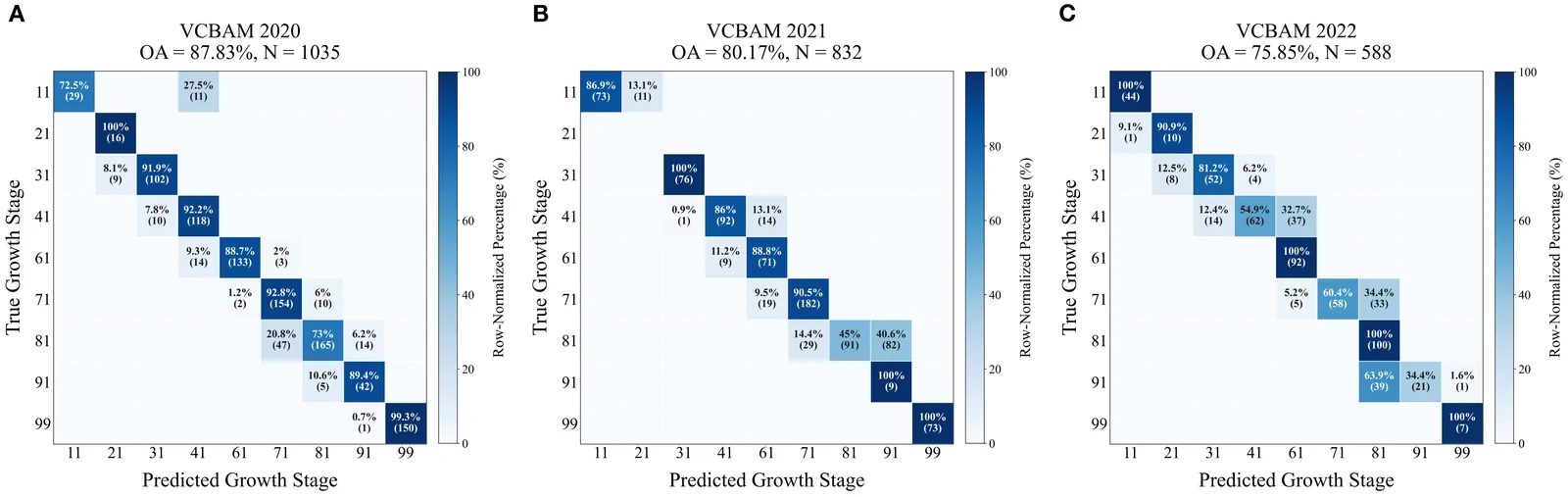
Row-normalized confusion matrices of VCBAM on the three independent cross-year test sets: **(a)** 2020; **(b)** 2021; and **(c)** 2022. Rows represent the true growth stages, and columns represent the predicted growth stages. Values indicate row-normalized percentages, and the sample counts of each true class are shown in parentheses.

To further examine the stage-wise prediction behavior of VCBAM under different cross-year conditions, row-normalized confusion matrices were generated for the three independent test years, as shown in **Figure 7**. The matrices present the prediction distribution across all nine maize growth stages. Overall, most samples were correctly classified along the diagonal, indicating that VCBAM maintained relatively stable stage-wise recognition performance across different test years. The main misclassifications occurred between adjacent or visually similar growth stages, which is consistent with the gradual and continuous morphological transitions of maize plants during development.

The year-specific differences in the confusion patterns can be partly explained by stage-boundary ambiguity and intra-class variation under field conditions. Maize growth is a continuous biological process, and samples collected near phenological transition periods may contain visual characteristics of adjacent stages. For example, Stage 81 in the 2021 independent test set showed relatively large intra-class variation.

Early-period samples of Stage 81 still retained green canopy structures and were visually close to Stage 71, whereas late-period samples showed stronger senescence-related characteristics and were visually closer to Stage 91. This broad transition range increased the visual ambiguity of Stage 81 and led to misclassifications toward adjacent stages for some models.

These results indicate that the year-dependent performance variation was not caused solely by a particular attention module, but was also closely related to the interaction between cross-year data distribution shifts and the visual ambiguity of transition-stage samples. Differences in illumination, image quality, soil exposure, canopy coverage, maturity progress, and background states across different test years may change the discriminative cues available for growth-stage recognition.

For a more complete class-wise comparison, the row-normalized confusion matrices of all compared attention mechanisms are provided in the **Supplementary Figure S3**. These supplementary results further illustrate the differences in prediction distributions among SE, ECA, CBAM, and VCBAM across the three independent test years.

### 3.3 Ablation study

#### 3.3.1 Module-level ablation

To evaluate the individual and combined contributions of the proposed components, eight ablation configurations were implemented based on the ResNet-50 backbone. These configurations include the baseline model, single-module variants with the TFM, VCBAM, and the MS Module, as well as different combinations of these modules. All configurations were evaluated on the three independent cross-year test sets from 2022, 2021, and 2020. The corresponding results are summarized in **Table 5**.

**Table 5:** Ablation study results of the proposed modules on the cross-year independent test sets.

Model	Components				Performance (%)						
	Base.	TFM	VCBAM	MS	OA-22	OA-21	OA-20	Mean OA	Macro-P	Macro-R	Macro-F1
ResNet-50	✓	×	×	×	73.47	79.33	84.73	79.18	76.74	76.56	73.45
TFM	✓	✓	×	×	78.51	75.36	83.48	79.11	76.5	76.2	73.1
VCBAM	✓	×	✓	×	75.85	80.29	**87.83**	81.32	80.43	82.18	77.68
Multi-Scale	✓	×	×	✓	76.7	74.16	87.05	79.3	78.98	81.07	75.76
TFM+VCBAM	✓	✓	✓	×	76.11	76.24	85.23	79.19	77.2	77.6	74
TFM+MS	✓	✓	×	✓	76.92	**81.85**	81.74	80.17	79.2	80.8	76.2
VCBAM+MS	✓	×	✓	✓	82.28	80.05	87.55	83.29	**82.6**	83.4	79.2
MS-TFNet	✓	✓	✓	✓	**84.69**	78.73	87.51	**83.64**	81.46	**83.77**	**79.73**

Base. denotes the ResNet-50 baseline; Macro-P, Macro-R, and Macro-F1 denote mean macro-precision, mean macro-recall, and mean macro-F1, respectively. Mean values are calculated by averaging the corresponding metrics over the three independent test years. Bold values indicate the best result in each performance column. The bold values indicate the best performance among all compared methods.

As shown in **Table 5**, the ResNet-50 baseline achieved a mean OA of 79.18%, a mean Macro-Precision of 76.74%, a mean Macro-Recall of 76.56%, and a mean Macro-F1 of 73.45% across the three independent test years. Among the single-module variants, VCBAM provided the most evident improvement, increasing the mean OA to 81.32%, the mean Macro-Precision to 80.43%, the mean Macro-Recall to 82.18%, and the mean Macro-F1 to 77.68%. This indicates that the vegetation-aware attention mechanism improves both overall classification accuracy and class-balanced recognition performance under cross-year field conditions.

The MS Module alone produced a slight improvement in mean OA, from 79.18% to 79.30%, while increasing the mean Macro-F1 from 73.45% to 75.76%. This suggests that multi-scale spatial representation is helpful for capturing maize plants with different sizes and structural characteristics. In contrast, TFM alone did not improve the mean OA compared with the baseline. Although TFM improved the OA on the 2022 test set, its performance decreased on the 2021 and 2020 test sets, indicating that acquisition-date information alone may be insufficient under cross-year phenological shifts.

When modules were combined, more evident improvements were observed. The combination of VCBAMand the MS Module achieved a mean OA of 83.29%, a mean Macro-Precision of 82.60%, a mean Macro-Recall of 83.40%, and a mean Macro-F1 of 79.20%. These results show that vegetation-aware attention and multi-scale feature extraction provide complementary effects. The complete MS-TFNet achieved the highest mean OA of 83.64%, the highest mean Macro-Recall of 83.77%, and the highest mean Macro-F1 of 79.73%, outperforming the ResNet-50 baseline by 4.46 percentage points in mean OA and 6.28 percentage points in mean Macro-F1.

Overall, the ablation results indicate that the proposed components contribute differently to model performance. VCBAM provides the strongest single-module improvement, the MS Module strengthens spatial representation, and TFM provides auxiliary phenological information when integrated into the complete framework. These results demonstrate that the final MS-TFNet benefits from the complementary integration of vegetation-aware attention, multi-scale visual representation, and temporal feature encoding.

#### 3.3.2 Stage-wise recognition performance analysis

**Figure 8:**
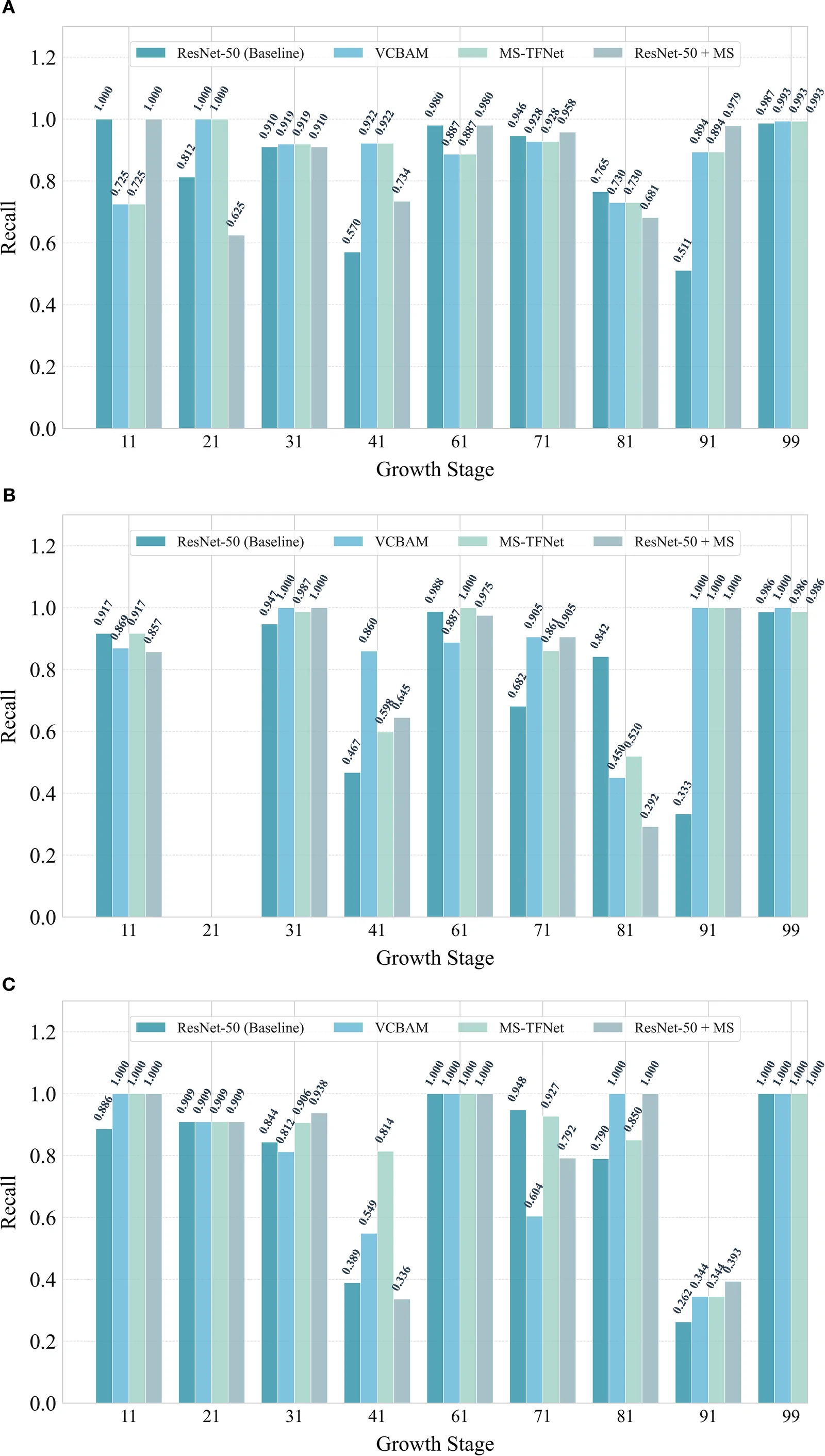
Stage-wise recall comparison of different models across three consecutive years. **(a)** Results for 2020; **(b)** Results for 2021; **(c)** Results for 2022.

To provide a more fine-grained evaluation of model behavior across different maize growth stages, stage-wise recall results over three consecutive years were analyzed, as shown in **Figure 8**. To complement the recall-based analysis, the corresponding stage-wise precision and F1-score results are provided in **Supplementary Figures S4** and **S5**, respectively.

The results show that the MS-TFNet exhibits improved consistency and stability across multiple growth stages. In particular, under early-stage conditions, stage transition periods, and visually ambiguous phenological stages, the model suppresses abnormal recall fluctuations and maintains stable performance across different years. This demonstrates that the proposed multi-scale structure and vegetation-enhanced attention mechanism enhance the robustness of discriminative feature extraction in complex field environments.

Due to the continuous nature of maize phenological development, adjacent growth stages often exhibit only subtle differences. For example, Stage 21 (Seedling Emergence) and Stage 31 (Three-leaf), as well as Stage 61 (Jointing) and Stage 71 (Tasseling), show gradual transitions in morphology that are difficult to distinguish. At these transition boundaries (e.g., 21 → 31, 61 → 71, and 81 → 91), which represent the most challenging scenarios, the MS-TFNet demonstrates more concentrated recall distributions and reduced inter-annual variability.

Overall, these results indicate that the proposed framework mitigates confusion between adjacent growth stages while maintaining recognition stability, aligning well with the continuous characteristics of maize growth.

#### 3.3.3 Temporal tolerance analysis

**Figure 9:**
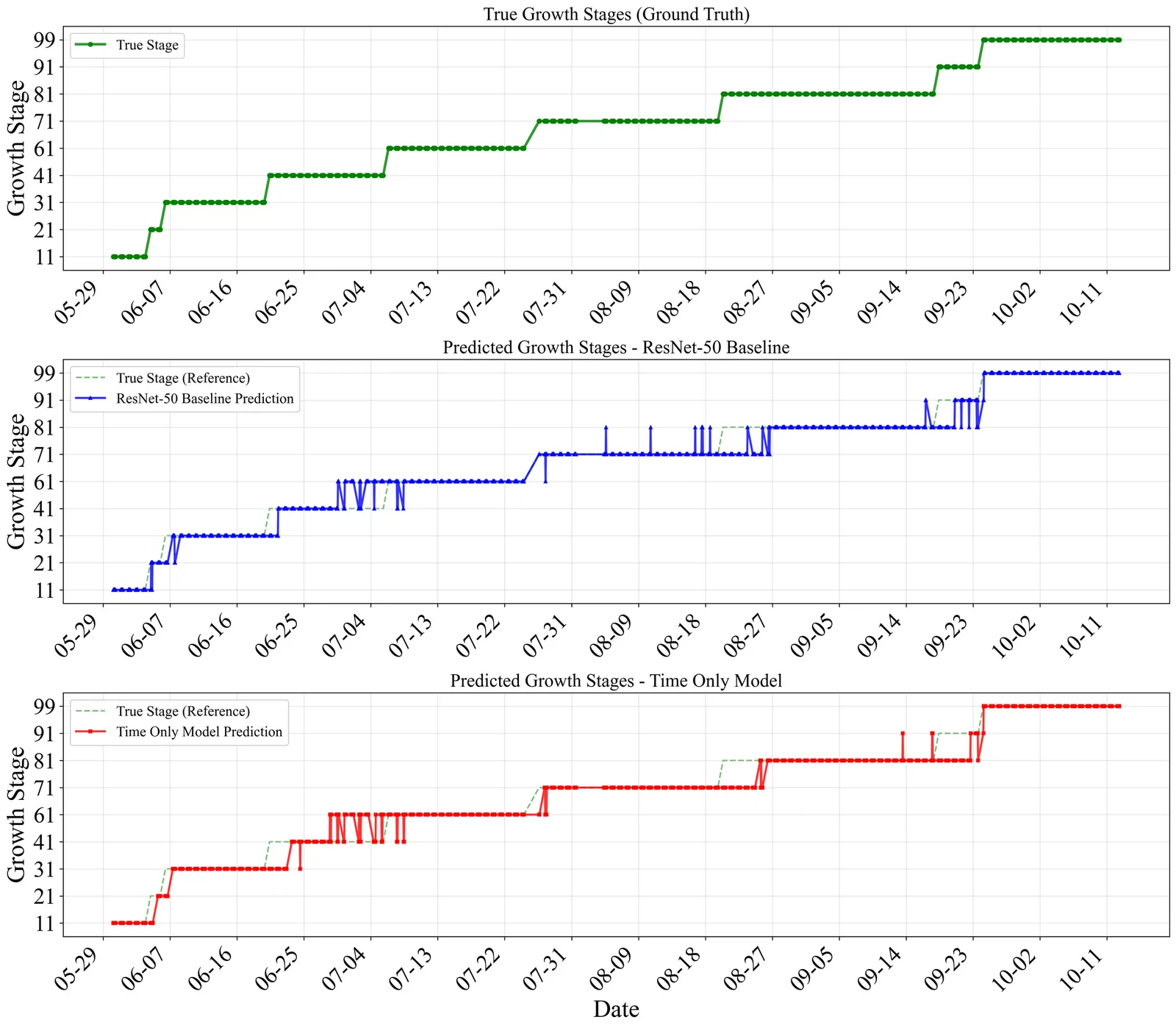
Comparison between ground-truth growth stages and model-predicted stage time series over a complete growing season in 2020.

Considering that maize growth follows a relatively regular developmental pattern under field conditions, temporal information serves as a critical constraint. To further evaluate the impact of temporal features on the temporal consistency of model predictions, **Figure 9** illustrates a comparison between the ground-truth growth stages and the model-predicted stage time series over a complete growing season in 2020.

The corresponding temporal prediction sequences for the 2021 and 2022 independent test sets are provided in **Supplementary Figures S6** and **S7**, respectively.

As illustrated in **Figure 9**, incorporating temporal features results in smoother predicted stage sequences with fewer abrupt transitions, indicating improved temporal consistency.

To further evaluate this property, a tolerance window of ±3 days was introduced, as shown in **Table 6**. The corresponding results show that the MS-TFNet achieves higher tolerant accuracy compared to the baseline, indicating improved alignment with the natural phenological progression.

**Table 6:** Recognition accuracy (%) of different models under a temporal tolerance of ± 3 days.

Model	2020 (%)	2021 (%)	2022 (%)	Mean (%)
ResNet-50	95.17	89.63	89.32	91.37
TFM	95.36	**92.70**	86.22	91.42
VCBAM+MS	96.04	90.99	87.76	91.60
MS-TFNet	**96.23**	91.35	**93.71**	**93.76**

The bold values indicate the best performance among all compared methods.

Under strict day-by-day evaluation, the MS-TFNet achieves an OA of 83.64%, outperforming the ResNet-50 baseline (79.18%) by 4.46 percentage points. The reduced performance gap under the tolerance criterion suggests that most baseline errors occur near stage transition boundaries as small temporal shifts rather than severe misclassifications.

Overall, the proposed model improves both stage discrimination and temporal stability, demonstrating enhanced spatio-temporal consistency for practical agricultural monitoring.

### 3.4 Comparison with recent studies and model interpretation

The experimental results show that MS-TFNet improved maize growth-stage recognition under independent cross-year field conditions by integrating the MS Module, TFM, and VCBAM. Compared with the ResNet-50 baseline, the complete model increased the mean OA from 79.18% to 83.64% and the mean Macro-F1 from 73.45% to 79.73%. The ablation results further indicate that the three proposed components play different but complementary roles in the model. Therefore, the performance improvement of the complete model does not result from a single module, but from the complementary integration of multiple types of information.

To further clarify the position of MS-TFNet in current maize growth-stage recognition studies, we compare it with recent crop phenology recognition studies. Ni et al. proposed MaizeHT, which combines a ResNet34 feature extractor with Transformer-based self-attention to integrate local convolutional features and global visual dependencies for maize growth-stage classification (Ni et al., 2024). The model achieved high classification accuracy on a self-built UAV RGB maize dataset. However, the dataset was collected from a single-year maize field and contained only four growth stages. In addition, 10,040 cropped images were randomly divided into training, validation, and test sets at a ratio of 8:1:1. Therefore, the reported results mainly reflect recognition performance under relatively consistent acquisition conditions and a limited number of growth stages. In contrast, MS-TFNet covers nine maize growth stages across eight growing seasons and is evaluated using independent station-year test subsets, emphasizing full-season coverage and generalization to unseen years and field conditions. Sun et al. recently proposed AMFR-Net for fine-grained wheat phenology recognition using ground-based RGB images (Sun et al., 2026). The model uses a ResNet-101 backbone and an adaptive multi-scale attention fusion module to improve the discrimination of visually similar phenological stages through cross-scale interaction and content-adaptive weighted aggregation. This finding is consistent with our result that combining the MS Module with VCBAM produced more evident performance improvement. However, AMFR-Net was mainly evaluated on an expert-labeled wheat subset using an 80:20 random split for a five-class classification task. In comparison, MS-TFNet has broader phenological coverage and a stricter cross-year evaluation setting. Methodologically, AMFR-Net emphasizes cross-scale interaction among hierarchical backbone features, whereas MS-TFNet uses parallel multi-scale convolutional branches and vegetation-aware feature refinement to enhance spatial representation at different receptive-field scales and crop-related feature selection. Li et al. introduced continuous environmental variables, including temperature, sunshine duration, soil moisture, and air humidity, into winter wheat growth-stage recognition based on multi-scale convolution and attention mechanisms (Li et al., 2025b). Their MMN-W model directly fused CNN-based image features with LSTM-based environmental sequence features, showing that environmental information can effectively complement visual features for distinguishing adjacent transition stages. However, this strategy requires continuous environmental monitoring and accurate synchronization between images and sensor data, and the dataset mainly came from a single experimental site during one winter wheat growing season. In contrast, MS-TFNet was evaluated under cross-year and cross-station conditions, and the acquisition date was not treated as a complete independent modality. Instead, it was used as a lightweight phenological prior fused with visual features, thereby maintaining the dominant role of visual evidence. Huang et al. proposed CornMFN, which uses cross-attention to fuse ConvNeXt image features, days after sowing, and multiple meteorological variables for corn phenological-stage identification (Huang et al., 2025). Their large-scale dataset covers multiple agroecological regions and complete corn growth stages, and their cross-year and cross-region experiments demonstrate the value of meteorological information for stage discrimination and geographical adaptability. However, this method relies on continuous meteorological observations, sowing-date records, and accurate synchronization between image and environmental data. In contrast, MS-TFNet uses only fixed-camera RGB images and acquisition dates, without requiring environmental sensors or complete time-series observations, and each image can be classified independently. Therefore, CornMFN is more suitable for multimodal monitoring scenarios with complete environmental data, whereas MS-TFNet focuses on long-term fixed-camera monitoring under lower input requirements. The experimental results further reveal the functional division and complementary effects of the components in MS-TFNet. The MS Module extracts spatial features from different receptive-field ranges through multi-scale convolutional branches and a dilated convolutional branch, which helps represent changes in plant size, canopy coverage, and structural complexity during maize development. VCBAM produced the most evident improvement among the single-module variants, indicating that crop-related feature refinement is important for natural field images. Grad-CAM visualization and the soil-background masking experiment also suggest that VCBAM can generate a more reasonable response distribution between crop regions and field contextual information. When VCBAM and the MS Module were combined, the model performance was clearly higher than that of either module alone, indicating the complementarity between multi-scale representation and attention-based refinement. TFM further provides a lightweight phenological prior through image acquisition dates, offering additional temporal context for growth-stage recognition with almost no extra data acquisition burden.

### 3.5 Limitations and future work

Several limitations should be considered when interpreting the current results. First, MS-TFNet uses only RGB images and acquisition dates, while environmental and management variables, such as temperature, solar radiation, precipitation, soil moisture, irrigation, and fertilization, are not included. These variables may provide additional information about crop development that cannot be directly observed from images alone. Future work will explore effective fusion of visual features and key environmental variables while considering the cost of data acquisition and synchronization.

Second, maize growth-stage labels are discrete representations of a continuous biological development process. Images collected near stage-transition boundaries may contain characteristics of two adjacent stages, increasing label ambiguity and classification uncertainty. Future studies may explore ordinal classification, soft labels, transition-stage modeling, or regression-based growth indicators to better represent the continuity of maize development.

Third, MS-TFNet does not explicitly model the dynamic relationships among consecutive observations. Existing studies have shown that sequence-based models, such as DenseNet–BiLSTM and CNN–LSTM, can use continuous images or multi-temporal remote sensing data to extract spatio-temporal variation information during crop development (Wang et al., 2025; Feng et al., 2025b). However, the dataset used in this study contains many temporally adjacent and visually highly correlated images, and some station–year trajectories are incomplete or imbalanced across stages. If sequence construction and data partitioning are not strictly controlled, the model may overfit to correlation patterns associated with specific stations, years, or imaging conditions. Therefore, future research should explore more robust sequence modeling methods based on more complete station–year trajectory data and stricter data partitioning strategies.

Finally, although the computational conditions of current field monitoring devices can support the deployment of the proposed model, model lightweighting remains important for larger-scale, multi-station, or edge-based real-time applications. Future work will investigate lightweight backbones, structured pruning, knowledge distillation, and deployment optimization for field monitoring systems.

## 4 Conclusions

This study proposed MS-TFNet, a maize growth stage recognition framework that integrates multi-scale feature extraction, vegetation-aware attention, and temporal feature encoding for RGB field images. The proposed framework was evaluated using independent cross-year test sets collected from different station-year conditions, aiming to assess its generalization ability under complex field environments.

The experimental results showed that MS-TFNet improved the overall cross-year recognition performance compared with the ResNet-50 baseline. The MS Module provided complementary spatial representations for maize plants with different target sizes and canopy structures. The proposed VCBAM improved the mean performance by refining vegetation-aware feature responses and strengthening maize-related visual representations. The TFM provided auxiliary phenological information, although its independent contribution to OA was limited.

Ablation experiments further demonstrated the complementary effects of the proposed modules. The combination of VCBAM and multi-scale produced a clear performance improvement, and the complete MS-TFNet achieved the highest mean OA and mean Macro-F1 among the tested configurations. These results indicate that integrating spatial multi-scale representation, vegetation-aware feature refinement, and temporal phenological cues can improve maize growth stage recognition under cross-year field conditions.

Despite these improvements, the current framework still relies mainly on RGB imagery and acquisition-date information. Environmental factors such as temperature, radiation, precipitation, soil moisture, and field management practices were not included. Future work will focus on integrating multi-source environmental data, improving the completeness and balance of cross-year datasets, and developing lightweight models for real-time field deployment.

## Data Availability

The datasets presented in this study can be found in online repositories. The names of the repository/repositories and accession number(s) can be found in the article/**Supplementary Material**.
