# Supplementary material for: MS-TFNet: a multi-scale temporal feature fusion network for maize growth stage recognition
Source: Front Plant Sci. 2026 Aug 21;17:1882260. doi: 10.3389/fpls.2026.1882260 (PMC13541695; doi:10.3389/fpls.2026.1882260)
Supplement: Supplementary file 1 [file DataSheet1.pdf]

Supplementary Material

1 SUPPLEMENTARY TABLE

Table S1. Mean macro-averaged performance metrics of different backbone networks across the three independent cross-year test sets.

Model	Mean OA (%)	Mean Macro-Precision (%)	Mean Macro-Recall (%)	Mean Macro-F1 (%)
VGG-16	77.22	77.51	73.93	70.12
VGG-16 Bn	75.68	66.13	70.04	64.68
VGG-19	79.02	75.15	75.18	71.79
VGG-19 Bn	77.39	77.06	76.54	72.18
ResNet-50	79.18	76.74	76.56	73.45
ResNet-101	79.03	81.04	78.04	75.02
MobileNetV2	78.56	77.02	71.77	69.13
MobileNetV3	73.42	73.28	69.71	65.64
EfficientNet-B0	63.74	71.15	66.53	61.79
ShuffleNetV1	76.96	78.34	75.71	72.47
ShuffleNetV2	78.29	77.84	71.48	68.40
DenseNet-121	78.85	71.34	69.73	67.13

Note: OA denotes overall accuracy. mean OA, mean Macro-Precision, mean Macro-Recall, and mean Macro-F1 were calculated by averaging the corresponding metrics over the 2020, 2021, and 2022 independent cross-year test sets.

2 SUPPLEMENTARY FIGURES

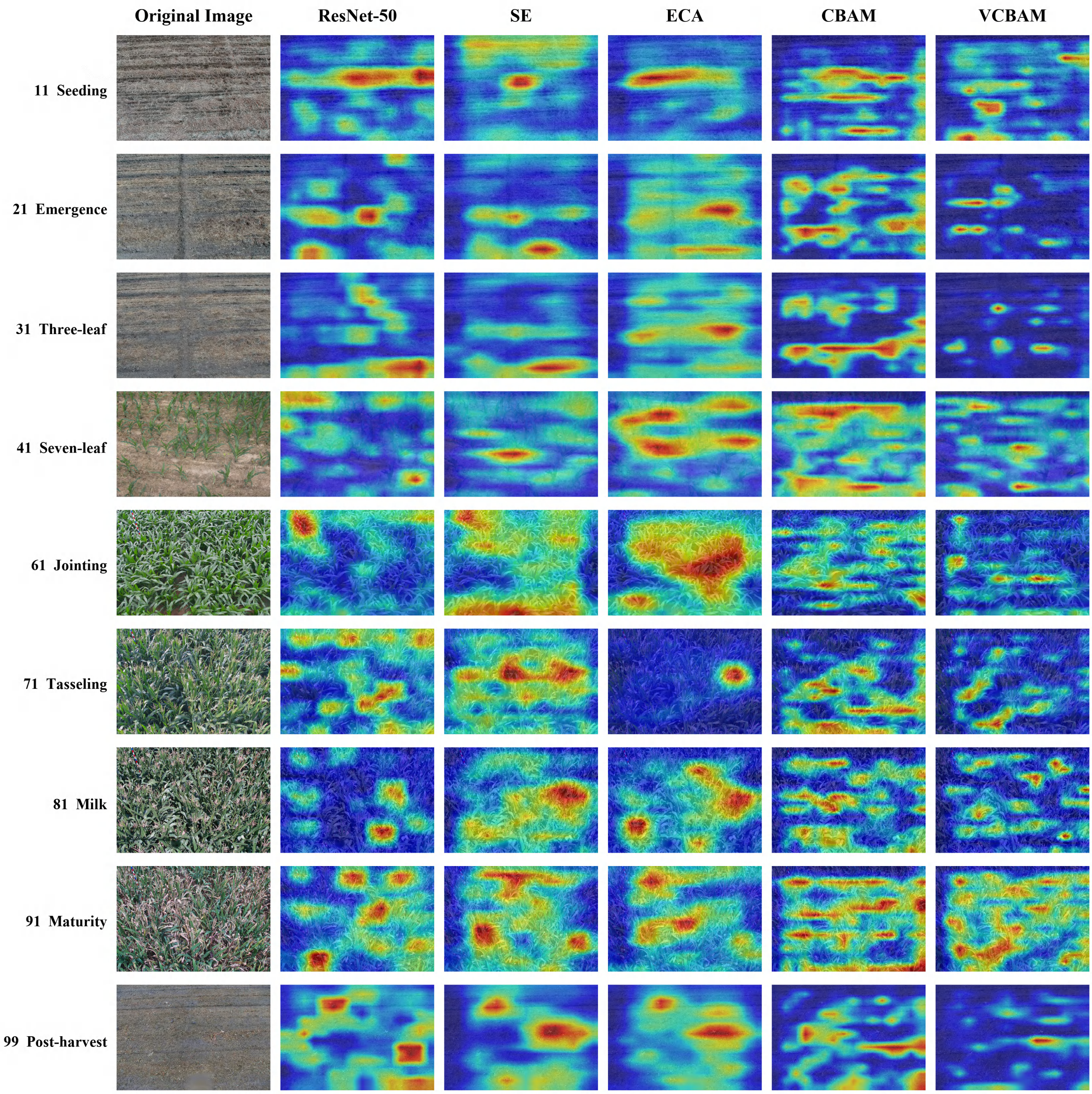

Figure S1. Stage-wise Grad-CAM visualization comparison across different maize growth stages and attention mechanisms. Rows represent maize growth stages, including Stage 11 to Stage 99, and columns represent the original image and Grad-CAM results of ResNet-50, SE, ECA, CBAM, and VCBAM. Warmer colors indicate regions with stronger model responses.

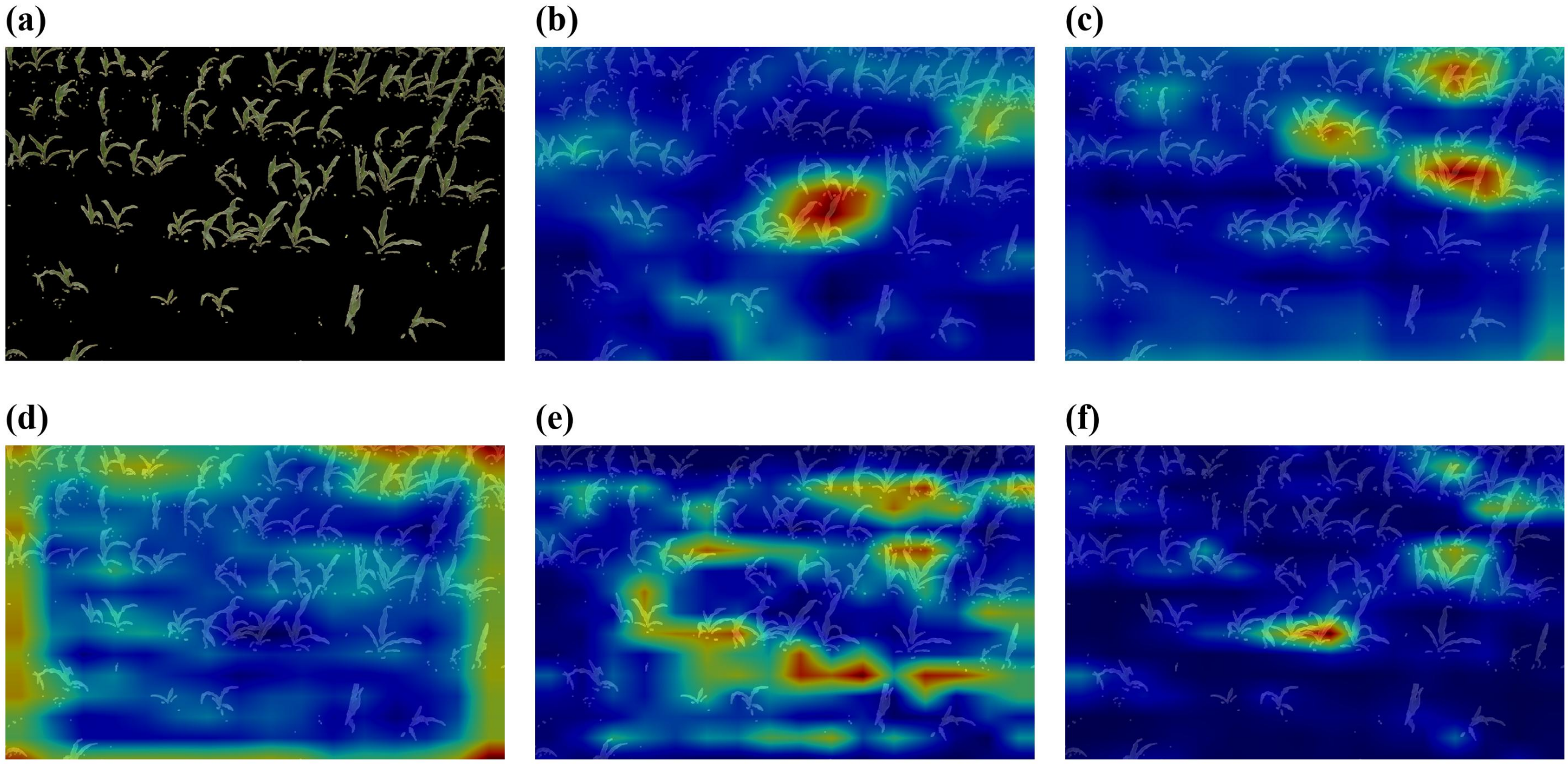

Figure S2. Soil-background masking analysis for Grad-CAM visualization. (a) Soil-masked image; (b) ResNet-50; (c) SE; (d) ECA; (e) CBAM; (f) VCBAM. Warmer colors indicate regions with stronger model responses.

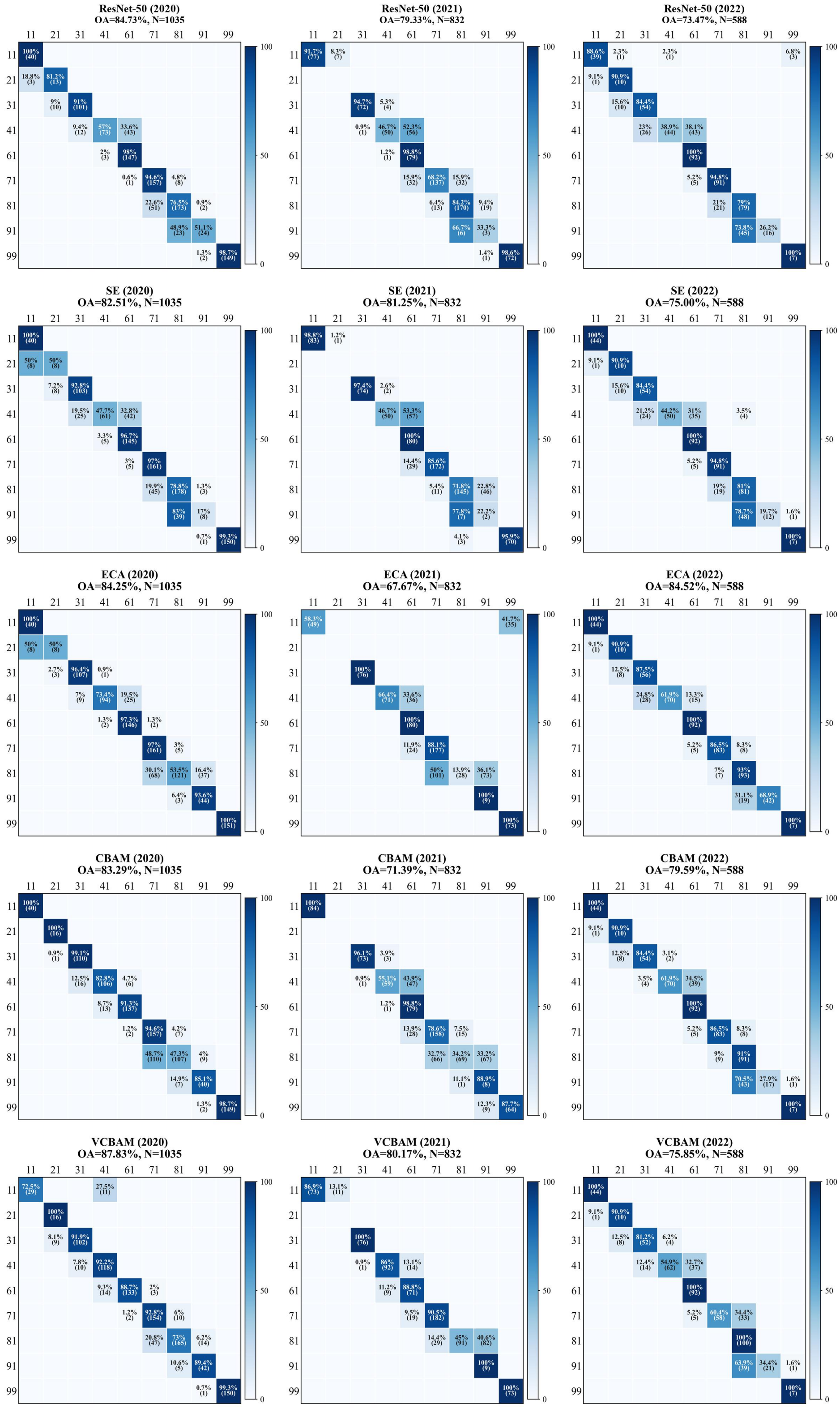

Figure S3. Confusion matrices of the compared attention mechanisms on the three independent cross-year test sets. Rows correspond to different models, including ResNet-50, SE, ECA, CBAM, and VCBAM. Columns correspond to the 2020, 2021, and 2022 independent test sets. In each confusion matrix, rows represent true growth stages and columns represent predicted growth stages. Values are row-normalized percentages, with sample counts shown in parentheses.

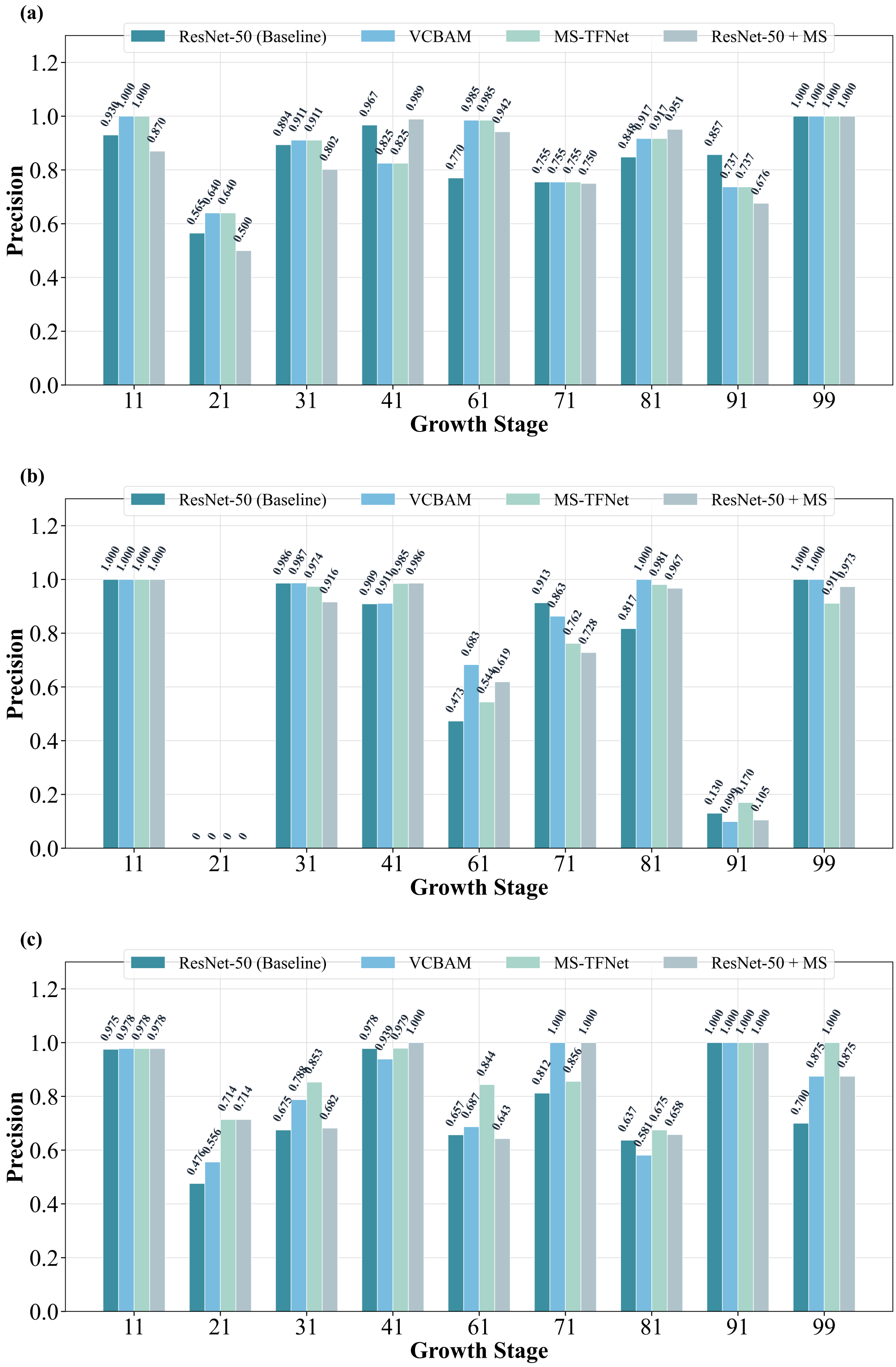

Figure S4. Stage-wise precision comparison of different model configurations across the three independent cross-year test sets. Subfigures (a), (b), and (c) correspond to the 2020, 2021, and 2022 independent test sets.

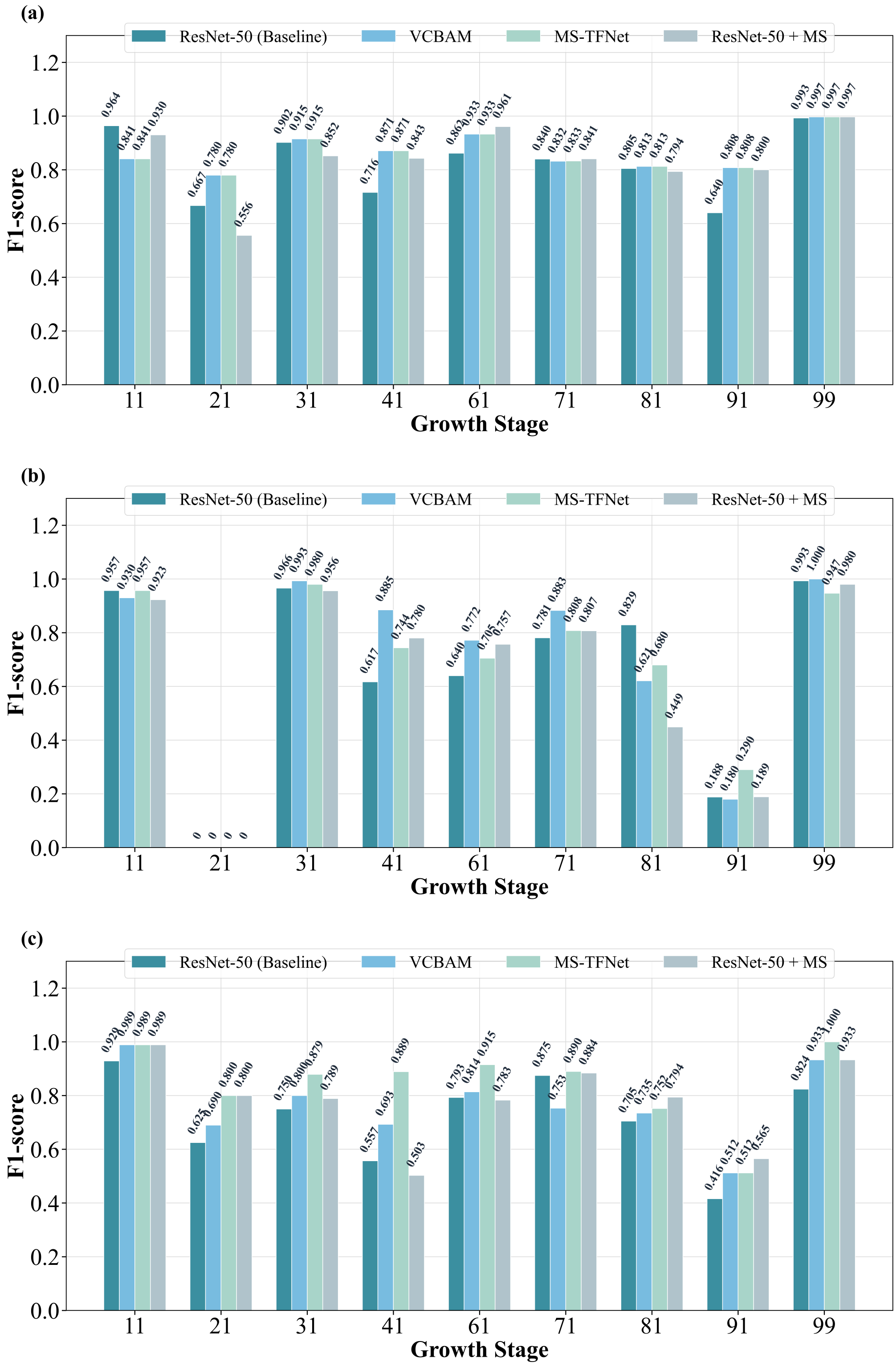

Figure S5. Stage-wise F1-score comparison of different model configurations across the three independent cross-year test sets. Subfigures (a), (b), and (c) correspond to the 2020, 2021, and 2022 independent test sets.

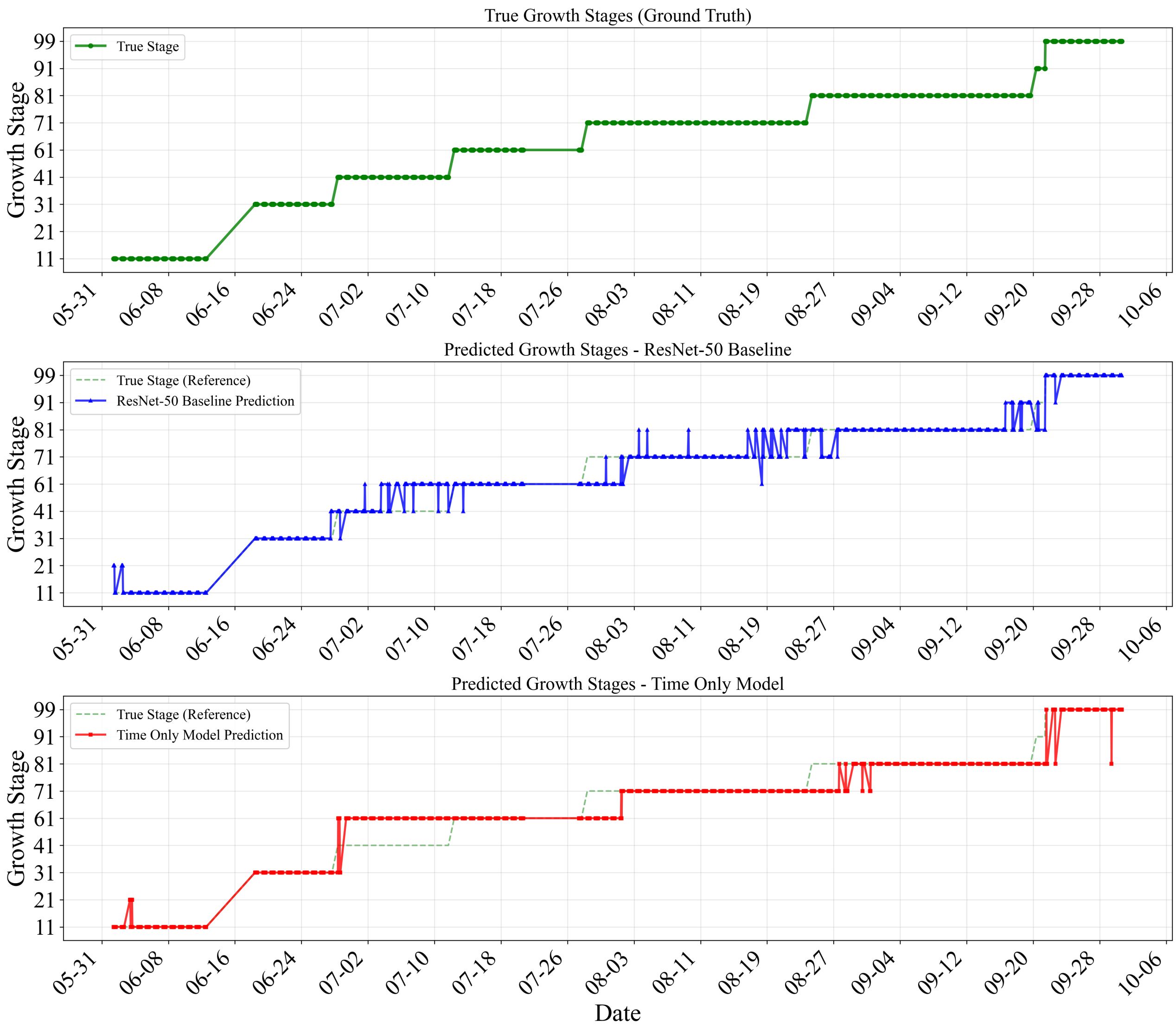

Figure S6. Temporal prediction sequence of growth stages in the 2021 independent test set. The upper panel shows the ground-truth growth-stage sequence, while the middle and lower panels compare the predicted stage sequences of the ResNet-50 baseline and the time-only model with the ground-truth reference, respectively.

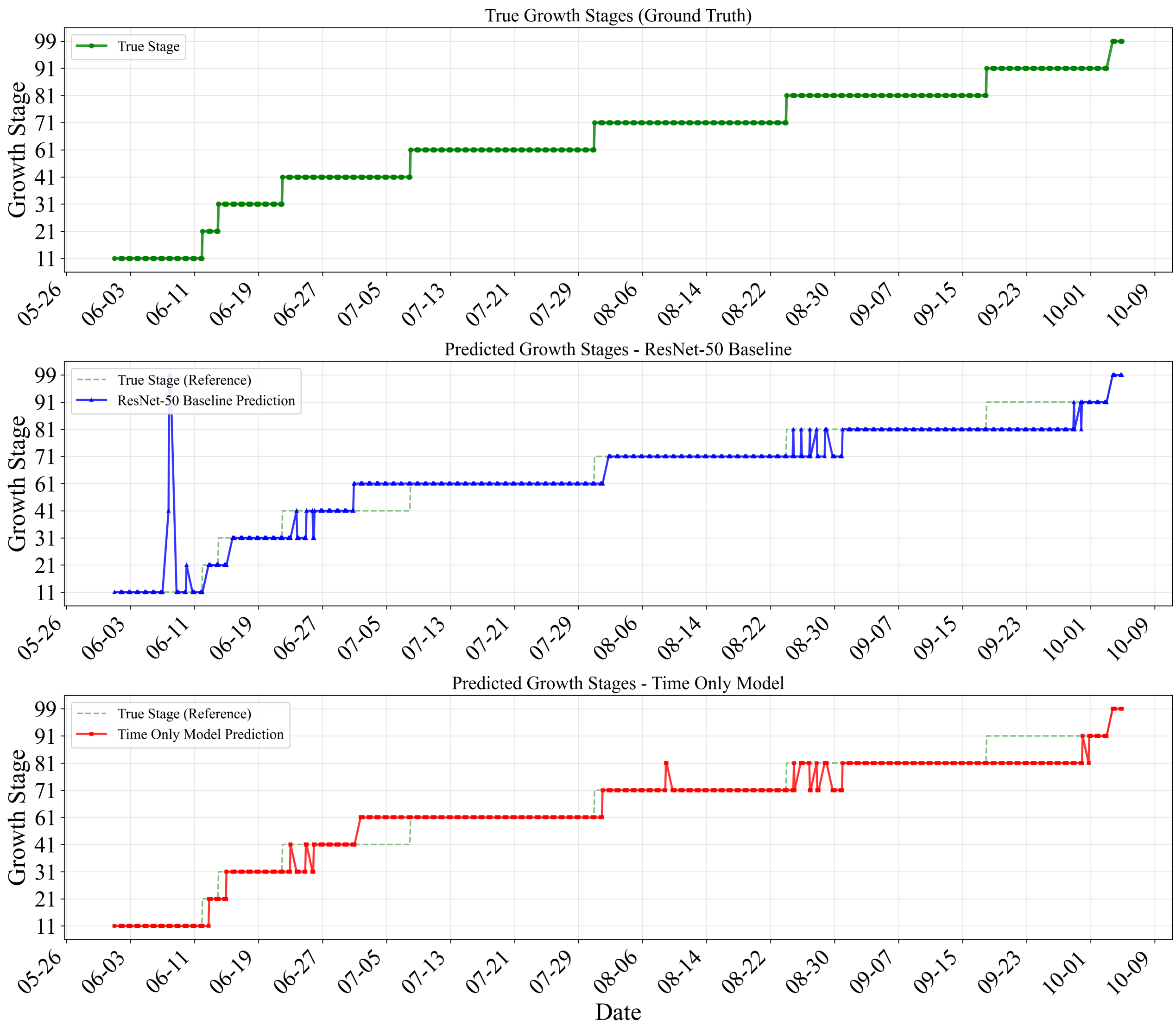

Figure S7. Temporal prediction sequence of growth stages in the 2022 independent test set. The upper panel shows the ground-truth growth-stage sequence, while the middle and lower panels compare the predicted stage sequences of the ResNet-50 baseline and the time-only model with the ground-truth reference, respectively.
